# 2019 Congress of the Italian Society of Neonatology: Catania, Italy. 25 -27 September 2019

**DOI:** 10.1186/s13052-019-0729-4

**Published:** 2019-12-18

## A1 Air pollutants and food contaminants: genetic and epigenetic consequences

### Ivana Antonucci, Alessandra Di Serafino, Prabin Upadhyaya, Luca Sorino, Liborio Stuppia

#### Department of Psychological, Health and Territorial Sciences, School of Medicine and Health Sciences, University “G. d’Annunzio”, via dei Vestini, 31, 66100, Chieti, Italy

##### **Correspondence:** Ivana Antonucci (i.antonucci@unich.it)

In recent years, it has been increasingly highlighted that nutrition plays a very important role in the modulation of gene regulation through epigenetic mechanisms (1). In particular, the presence of endocrine disruptors in food can have serious repercussions not only on the individual's health status, but also on their own children and even on their grandchildren (2). Indeed, it has been shown that an incorrect diet can induce epigenetic modifications on male and female gametes capable of causing dysregulations of gene expression in offspring with an increased risk of diseases such as obesity, diabetes and hypertension (3). In this scenario, it is important to underline that the same obesity induces epigenetic modifications in the spermatozoa of subjects with high BMI, which therefore will have a risk of children more susceptible to “chronic non-communicable diseases” (4). Epigenetic mechanisms are also implicated during *in utero* development, so environmental exposures may affect the fetus by impairing the epigenome of the developing organism to modify disease risk later in life (1). There is therefore clear evidence that epigenetic modifications are responsible not only for an individual risk linked to exposure to harmful environmental factors and to incorrect nutrition, but even to a transgenerational risk that could unpredictably damage the health of future generations (4). For this reason, a constant and targeted recourse to a natural and substance-free diet capable of inducing epimutations is an essential requirement for the primary prevention of the health of the new generations (5).

**Acknowledgements**

This study was supported by grants from the Italian Minister of University and Research (MIUR) 2015 prot. 20157FF4KM_002 to Liborio Stuppia.

**References**

1. Tiffon C. The impact of nutrition and environmental epigenetics on human health and disease. International Journal of Molecular Sciences. 2018.

2. Darbre PD. Endocrine Disruptors and Obesity. Current obesity reports. 2017.

3. Ling C, Rönn T. Epigenetics in Human Obesity and Type 2 Diabetes. Cell Metabolism. 2019.

4. Franzago M, Fraticelli F, Stuppia L, Vitacolonna E. Nutrigenetics, epigenetics and gestational diabetes: consequences in mother and child. Epigenetics. 2019.

5. Ideraabdullah FY, Zeisel SH. Dietary Modulation of the Epigenome. Physiol Rev. 2018;

## A2 Neurodevelopmental outcome of preterm infants of less than 32 weeks gestation: A 6-year retrospective cohort study of the Marche Neonatal Network

### B. Bartolomei^1,2^, E. Ferretti^1^, A. Peretti^1^, F. De Angelis^1,2^, C. Proietti Pannunzi^1,2^, R. D’Ascenzo^1^, V. P. Carnielli^1,2^

#### ^1^Salesi Children's Hospital, Ancona, Italy; ^2^Polytechnical University of Marche, Ancona, Italy

##### **Correspondence:** B. Bartolomei (bartolomeibeatrice@gmail.com)

**Introduction**: Neurodevelopmental impairment is a major long-term complication for a variable number of extremely low gestation neonates (ELGAN). Rates of survival have increased during the past 2 decades however neuromotor disabilities have not decreased.

**Objective**: The aim of this study was to obtain neurodevelopmental disabilities at 2 years corrected age of infants born in the Marche Region between 24^0/7^ and 31^6/7^weeks’ gestation from 2010 to 2016.

**Methods/Design**: Retrospective evaluation of two-year follow-up data prospectively collected for all infants born at 24^0/7^ to 31^6/7^ weeks’ gestation admitted to the NICU of the Salesi Children’s Hospital, Ancona, from January 2010 to December 2016. Exclusion criteria were major congenital malformations and admission after 48 hours of life. Cognitive (from 2010), motor (from 2010) and language (from 2014) development were assessed with Bayley Scales of Infant and Toddler Development 3rd edition and cerebral palsy was graded with Gross Motor Function Classification System (GMFCS). Visual and hearing data were also collected. Moderate and severe impairment (NDI) was defined as: a GMFCS ≥3, unilateral or bilateral blindness, hearing loss improved or not by aids or a developmental score ≤2 SDS.

**Results**: 630 infants admitted to our NICU in the study period were included. Forty-four (6.9%) died before 24 months of age. Five hundred eighty-six infants were eligible for the follow up. Eighty-eight (15%) were lost (missing data, failed to bring their children/moved away, declined the invitation, untraceable to the telephone call). 498 infants were available for the 2y follow-up. Birth weight was 1194 g ± 356; mean GA 204 days ± 14, 258 (52%) were males. Infants with extreme prematurity (24^0/7^-27^6/7^) were 132 and 366 were very preterm (28^0/7^-31^6/7^).

Mean cognitive composite (n=489) score was 94±14, motor score (n=490) 101±13 and language score (n=151) 96±18. Mild visual impairment was present in 15 infants (3%). Any hearing impairment was present in 3 (0.6%), with only 1 severe (bilateral deafness not improved by aids).

Cerebral Palsy was diagnosed in 15 infants (3.2%): moderate (GMFCS 3) in 4 (0.8%) and severe (GMFCS 4-5) in 7 (1.4%). Moderate and severe NDI was found in 32 infants (6.4%): 13 extreme preterms and 19 very preterms. Birth weight and gender were significantly associated with overall neurodevelopmental impairment (p=0.030, p=0.002 and p<0.001 respectively), but only gender was associated with moderate-severe outcome (p=0.026).

**Conclusion**: Preterm infants of the Marche Neonatal Network cohort born from 2010 to 2016 had relative low rate of moderate-severe impairment at 2 years corrected age in comparison with published data from similar gestational age groups. Follow-up at school age is much needed.

## A3 Neonatal pain and indicator validity

### Fabiola Bontempo, Federica Logrippo, Mattia Luciano, Maria Chiara Ariotti

#### Neonatal Intensive Care Unit, City of Health and Science – University of Turin, Turin, Italy

##### **Correspondence:** Fabiola Bontempo (fabiola.bontempo95@gmail.com)

**Background**

In 2008 in Italy, 2nd edition of Neonatal pain prevention and management guidelines [1] have been released, but they haven’t been adopted yet into practice. The A.O.U. City of Health and Science of Turin produced an operating instruction named *“preterm and term infants pain assessment and pain relief”* to make medical-nursing clinical behaviors uniform and improve the quality of assistance to infant with pain.

**Materials and methods**

The aim is to evaluate the impact of the operating instruction *“preterm and term infants pain assessment and pain relief”.* Sant’Anna Hospital NICUs (City of Health and Science of Turin) adopted the operating instruction and medical-nursing staff attended an educational session about it. The Quality Indicators (QIs) were monitored (use of saccharose to prevent procedural pain, pharmacological pain treatment, pain assessment at least 3 times a day) and the standards achievement was verified. Methods used to monitor QIs were: analysis of 30 infants’ medical records for 12 months, observations of nurses’ activity for 6 months and a questionnaire undergone to nurses to complete the research. The study group was composed by quality improvement experts and members of NICUs’ pain management team.

**Results**

Medical records analysis showed that the standard about procedural pain prevention with saccharose was not achieved, while the observations of nurses’ activity turned out that the saccharose was used in most procedural invasive interventions (>70%). These discording data suggested that QIs used were ineffective to monitor the real standards achievement. Questionnaire analysis added that nurses have difficulties to record their interventions about procedural pain prevention and pain assessment. Nurses must implement their knowledge about pain evaluation and management.

**Conclusions**

The use of operating instruction *“preterm and term infants pain assessment and pain relief”* played a key role in improving pain management in the NICUs. However, the results bring about the study group to introduce corrective actions: operating instruction review, QIs implementation, medical record review and a new educational session.

1. Lago P, Merazzi D, Garetti E, Ancora G, Pieragostini L, Bellieni CV, et al. Linee guida per la prevenzione ed il trattamento del dolore nel neonato. Gruppo di studio di analgesia e sedazione nel neonato. Società Italiana di Neonatologia. Biomedia s.r.l. 2016

## A4 Hashtag: I’ m standing with NICU of Spedali Civili Brescia

### Laura Bruno, Porta Scarta Elena, Elisabetta Dioni

#### Neonatal Intensive Care Unit, Hospital of Children, ASST Spedali Civili, Brescia, Italy

##### **Correspondence:** Laura Bruno (laurabruno.92@gmail.com)

**Background**

The Spedali Civili's instance blasted in July 2018 when the main TV channels and newspapers reported the death in our unit of a premature baby caused by Serratia Marcescens infection. Immediately after, infections control procedures were put into practice, successfully preventing the spread of the bacteria. Media misinformation though, continued, provoking a chain reaction on social sites that caused suspicion and mistrust towards the NICU health professionals and the entire hospital. The current widespread access to communicating tools and social media undoubtedly gave all new chances and opportunities to share information, comments and viewpoints; on the other hand if improperly used, it can lead to serious damages.

**Case report**

In January 2019, the concurrent death in our unit of three premature babies was loudly proclaimed by the main media, spreading the shadow of an epidemic linked to incompetent health service. What followed both on main TV channels and social media, was a cruel and offensive assault towards NICU health workers that generated fear and scepticism. The mentioned facts impacted strongly on the working environment; emotional tension grew between the staff and the patients' parents, making this important relationship laborious. NICU team remained united continuing to support and reassure constantly the parents. Critical thinking and self analysis were also carried on aiming to improvements. After few days of a non stop flow of outrageous comments, a group of nurses decided to react and answered back using the same attacking channel; in these posts was underlined the importance to shield the nursing profession while respecting the pain of those parents who lost their baby. The #IOSTOCONLATINDELCIVILE was created and spread, gaining huge support both from health careers and from parents who had lived the reality of our unity.

**Conclusion**

Strict guidelines are available regarding the proper use of social media by nurses and health workers; with this presentation though, we would like to urge more focus and consideration by professional groups and health organization in order to protect and value the nursing profession on social media.

Acknowledgements to Ardesi Marzia, Cavagna Federica, Gallini Laura, Guarinoni Luisa, Romitti Mariagrazia.

## A5 Maternal milk: news

### Maurizio Carta, Giovanni Corsello

#### Neonatal Intensive Care Unit, AOUP “P. Giaccone” Department of Health Promotion Sciences Maternal and Infantile Care, Internal Medicine and Medical Specialities "G. D'Alessandro", University of Palermo, Palermo, Italy

##### **Correspondence:** Maurizio Carta (mauriziocarta@yahoo.it)

For infants, human milk (HM) should be the only food in the first six months of life. HM has a strong health-promoting effect on both the mother and the infant and supplies not only all the essential nutrients but also bioactive compounds. The last two years have resulted in pivotal studies that clearly confirm the importance of breastfeeding for infants' short-term health with respect to growth, immune and gastrointestinal function. In addition, breastfeeding gives potential long-term advantages in term of malignant, metabolic and allergic diseases.

Breastfeeding enhances human capital by increasing intelligence. Some breastmilk factors promote maturation of the corpus callosum and cerebral cortical gray matter and are related to improved structural connectivity of developing networks. Certain type of cells in breast milk display many properties typical of stem cells and highlight the possibility of new therapeutic approaches. Moreover, specific class of breast milk lipid mediators are essential for healthy adipose tissue development: alkylglycerols contribute to maintain beige adipose tissue (BeAT) phenotype in infants preventing transformation of BeAT in white adipose tissue. We also know that exclusive breastfeeding has a great impact on shaping the infant's gut microbiota and these findings are important for allergy and infection prevention.

The last reports highlight the importance of breastfeeding practices. Some studies suggest that milk given via breast pumps is found to contain high numbers of harmful bacteria. Some clinical studies supported the enormous progress in human milk oligosaccharides (HMOs) research. HM administration after bone marrow transplant is safe in young children and might reduce intestinal inflammation. Breastfeeding is clearly relevant for non-communicable diseases such as breast cancer, obesity and diabetes representing a cost-effective risk reduction strategy for these aggressive diseases.

All studies confirm that breastfeeding permanently shapes individuals' life course. Although there is no doubt regarding the multiple benefits of breastfeeding for infants and society, in the last year a critical evaluation of the current ten steps to successful breastfeeding was completed because of emerging controversy regarding its efficacy and safety. Each Institution must adapt its program according to their current practices and perceptions.

## A6 Neuro Critical Care

### Giorgio Cautadella (gioblues71@gmail.com)

#### UTIN Ospedale S .Marco – Catania, Italy

Neuro Critical Care is an emerging care model involving an "inter-professional team born to recognize early and deal with adverse neurological conditions of preterm and full term babies in NICUs

The Inter-professional Expert Team is composed of Neonatologist, Pediatric Neurologist, Nurse and EEG technician (where and present).

The objectives:

- Early recognition and treatment of adverse neurological conditions
- Neurological assistance with group interprofessional approach
- Prevention of Secondary Brain Injuries
- Support and assistance for long-term inpatients through long-term follow-up

The nurse’s key role is continuous assistance in “BedSide” with Neurological observation and evaluation, and Application (in the absence of the EEG technician) of neurophysiological aEEG monitoring. safe method well tolerated to monitor electrocortical activity, then neonatal brain function by providing useful information on short and long term prognosis.

## A7 Continuous infusion of vancomycin in the treatment of neonatal sepsis: pharmacokinetics, efficacy and safety profile

### Elisa Cavalleri^1^, Marco Ginammi^1^, Giada Gennati^1^, Roberto Marzollo^1^, Mario Regazzi^2^, Gaetano Chirico^1^

#### ^1^Neonatal Intensive Care Unit, Children's Hospital, ASST Spedali Civili, Brescia, Italy; ^2^Società Italiana di Farmacocinetica e Biofarmaceutica (S.I.F.E.B.), Pavia, Italy

##### **Correspondence:** Elisa Cavalleri (elicavalleri@gmail.com)

**Background**

Vancomycin is a time-dependent bactericidal glycopeptide used in the treatment of neonatal sepsis, especially in the late-onset forms. In literature there are few data regarding its safety profile in neonatal settings, and a precise therapeutic target has not yet been defined.

**Materials and methods**

The aim of our study was to evaluate pharmacokinetics, safety profile and possible adverse effects related to continuous infusion of vancomycin in newborns.

We have retrospectively evaluated 54 patients admitted to our Neonatal Intensive Care Unit (February 2016 - December 2018) for a total of 66 cycles of therapy and 129 plasma dosages. The daily dose was calculated based on post-natal and post-menstrual age according to previous studies [1,2,3,4]. At about 48-72 h after treatment starting, plasma dosage was performed with subsequent possible change in the rate of infusion. Dose reduction was planned for patients with renal insufficiency. Creatinine clearance (Clcr) was evaluated with the Schwartz formula. Clearance of vancomycin (CLvanc) was calculated using the equation [Ro(mg/h)/Css(mg/L)]/body weight(kg), where Ro represents the infusion rate and Css the average concentration at steady state. A p value ≤0.05 was considered statistically significant. The audiometric function was evaluated by otoemission, automatic evoked acoustic trunk potentials or by clinical audiological examination.

**Results**

Mean birth weight was 1197 g (433-3560), and gestational age 28.7 weeks (22.5-40.3). Fifty-one patients began sepsis treatment with continuous infusion vancomycin preceded (91%) by a loading dose (79.6%: 13-16 mg/kg). In 60.6% of cases the vancomycinaemia was within the therapeutic range (10-25 mg/l) at the first plasma dosage. Correlations of Clcr with gestational age or birth weight were statistically significant. Mean total CLvanc at the steady-state for all measurements was 0.069 l/h/kg (0.053-0.091), with a significant increase related to postnatal age. A significant correlation between Clcr and CLvanc was observed; however, CLvanc was not influenced by post-natal age. Neither nephrotoxic effects nor correlations with alterations in audiometric tests were recorded.

**Conclusions**

Despite the limitations related to the retrospectivity of the study, the poor homogeneity of the sample and the variability of dose and of plasma dosing times of vancomycin, our study confirms the few data available in terms of feasibility and safety. The CLvanc and Clcr significant correlation is also confirmed, therefore, it seems possible to use the Clcr to establish the vancomycin dose, while waiting the vancomycinaemia. Some aspects remain to be clarified regarding the 24-hour-dose and timing to reach therapeutic concentration after the loading dose.

**References**

1. Zhao W, Lopez E, Biran V, Durrmeyer X, Fakhoury M, Jacqz-Aigrain E. Vancomycin continuous infusion in neonates: dosing optimization and therapeutic drug monitoring. Arch Dis Child. 2013; 98:449-453.

2. Plan O, Cambonie G, Barbotte E, Meyer P, Devine C, Milesi C, Pidoux O, Badr M, Picaud JC. Continuous infusion vancomycin therapy for preterm neonates with suspected or documented Gram-positive infections: a new dosage schedule. Arch Dis Child Fetal Neonatal Ed. 2008; 93:418-421.

3. Oudin C, Vialet R, Boulamery A, Martin C, Simon N. Vancomycin prescription in neonates and young infants: toward a simplified dosage. Arch Dis Child Fetal Neonatal Ed. 2011; 96:365-370.

4. Pawlotsky F, Thomas A, Kergueris MF, Debillon T, Roze JC. Constant rate infusion of vancomycin in premature neonates: a new dosage schedule. Br J Clin Pharmacol. 1998; 46:163-167.

## A8 Prevention and management of hypoglycemia

### Gaetano Chirico (gaechirico@alice.it)

#### U.O. di Neonatologia e Terapia Intensiva Neonatale, Presidio Ospedale dei Bambini, ASST Spedali Civili di Brescia, Italy

**Background**

Recent studies confirmed the risk of long term sequelae after neonatal hypoglycemia [1,2]. It is of paramount importance the prevention, diagnosis and treatment of hypoglycemia, particularly in infants admitted to first level units. The serum level cutoff is still matter of debate, however, the most frequently level considered during the first 48 hours of life is <45 mg/dL [3], while after it may considered <50 mg/dL according to AAP, or <60 mg/dL for PES [4].

Main factors favoring neonatal hypoglycemia are reduced glucose supply, low glycogen or fat stores, inadequate ratio between glucose production and consumption, endocrine imbalance or impairment, and some maternal drugs [5].

The most frequent high-risk populations in first level units are those large (LGA), or small for gestational age (SGA), infants of diabetic mother (DID), and late preterm infants (LPI). Other risk infants are discordant birth weight twins, those born after perinatal stress, or those affected by congenital endocrine or metabolic disorders, particularly when associated to hyperinsulinism [5]. An overall incidence of 50% of hypoglycemia has been reported in these high-risk infants.

The main symptoms may be sweating, feeding difficulties, tremors, irritability, weak or high-pitched cry, seizures, hypothermia, lethargy, hypotonia, or cyanosis [5].

We analyzed the LGA, SGA, DID, and LPI high-risk infants groups in our nursery during the year 2018. Out of the 416 evaluated newborns, 135 (32.5%) showed values <45 mg/d, with the highest incidence observed in late preterm infants (55.6%). In a previous study [6] the global incidence was lower (17.8%), however, a different cutoff of 40 mg/dL was adopted during the first 4 hours. Similarly to our findings, late preterm infants had the uppermost incidence (34%).

**Conclusion**

These data confirm the well-known evidence that hypoglycemia is the most frequent metabolic complication during the neonatal period, and that the late preterm infants are at the highest risk. It is, therefore, required to adopt appropriate protocols for the management of hypoglycemia in high-risk infants, providing frequent blood levels measurements, particularly during the first six hours. Continuous glucose monitoring is under investigation [7]. The very early breast-feeding or, if not available, formula feeding, should always be provided, while the supplementation with formula, or dextrose gel [8, 9, 10] is necessary when hypoglycemia is confirmed. When the infant is symptomatic, or the hypoglycemia is persistent, parenteral glucose administration is mandatory, and admission to the NICU is suggested. In infants with the most severe hypoglycemia, or with congenital disorders, the risk of persistent hypoglycemia should be excluded before discharge [11].

**References**

1. McKinlay CJD, Alsweiler JM, Anstice NS, et al. Association of neonatal glycemia with neurodevelopmental outcomes at 4.5 years. JAMA Pediatr 2017; 171:972–83.

2. Shah R, Harding J, Brown J, McKinlay C. Neonatal Glycaemia and Neurodevelopmental Outcomes: A Systematic Review and Meta-Analysis. Neonatology. 2019; 115:116-126.

3.. Committee on Fetus and Newborn, Adamkin DH. Postnatal glucose homeostasis in late-preterm and term infants. Pediatrics. 2011; 127:575-9.

4. Thornton PS, Stanley CA, De Leon DD, et al. Pediatric Endocrine Society. Recommendations from the Pediatric Endocrine Society for Evaluation and Management of Persistent Hypoglycemia in Neonates, Infants, and Children. J Pediatr. 2015; 167:238-45.

5. Abramowski A, Hamdan AH. Neonatal Hypoglycemia. 2019 Jan 25. StatPearls. Treasure Island (FL): StatPearls Publishing; 2019 Jan-. Available from http://www.ncbi.nlm.nih.gov/books/NBK537105/

6. Hosagasi NH, Aydin M, Zenciroglu A, Ustun N, Beken S. Incidence of hypoglycemia in newborns at risk and an audit of the 2011 American academy of pediatrics guideline for hypoglycemia. Pediatr Neonatol. 2018; 59:368-374.

7. McKinlay CJD, Chase JG, Dickson J, Harris DL, Alsweiler JM, Harding JE. Continuous glucose monitoring in neonates: a review. Matern Health Neonatol Perinatol. 20173 :18.

8. Harris DL, Weston PJ, Signal M, Chase JG, Harding JE. Dextrose gel for neonatal hypoglycaemia (the Sugar Babies Study): a randomised, double-blind, placebo-controlled trial. Lancet. 2013; 382:2077-83.

9. Glasgow MJ, Harding JE, Edlin R; Children with Hypoglycemia and Their Later Development (CHYLD) Study Team. Cost Analysis of Treating Neonatal Hypoglycemia with Dextrose Gel. J Pediatr. 2018; 198:151-155.

10. Coors SM, Cousin JJ, Hagan JL, Kaiser JR. Prophylactic Dextrose Gel Does Not Prevent Neonatal Hypoglycemia: A Quasi-Experimental Pilot Study. J Pediatr. 2018; 198:156-161.

11. Rozance PJ, Wolfsdorf JI. Hypoglycemia in the Newborn. Pediatr Clin North Am. 2019; 66:333-342.

## A9 Nutrition and growth aspects of the very preterm newborn

### Alessandra Coscia^1^, Chiara Peila^2^, Paola Di Nicola^3^, Giulia Maiocco^1^, Francesco Cresi^1^

#### ^1^Neonatal Unit, Department of Public Health and Pediatric, University of Turin, Turin, Italy; ^2^Department of Maternal and Neonatal Medicine, C. Arrigo Children's hospital, Alessandria, Italy; ^3^Neonatal Unit, Santa Croce e Carle Hospital, Cuneo, Italy

##### **Correspondence:** Alessandra Coscia (alessandra.coscia@unito.it)

Preterm VLBW infants are at risk of short- and long-term growth failure. This risk is inversely proportional to gestational age and is linked to several factors, some of which are prenatal (in particular the extent of intrauterine growth retardation, IUGR), and some occur during the hospital stay in NICU: this period is particularly influenced by severity of morbidity (NEC, feeding intolerance, feeding difficulties, respiratory distress, late onset sepsis, severe IVH, bronchodysplasia).

For the above-mentioned reasons, the goal of assuring a postnatal growth rate that approximates the intrauterine growth rate is far from been achieved. Therefore, the majority of VLBW infants experience a certain degree of extrauterine growth restriction (EUGR).

The post-discharge growth pattern is peculiar. At 2-3 years of age, approximately 80% of VLBW preterm infants have normal weight and height values, but on average they are lower than those of their full term pairs, despite the high catch up growth [1]. As reported by large international observational studies [2], part of these subjects recover even later, between 2 and 5 years of age. Late catch up growth is a characteristic of preterm babies with fetal growth restriction in particular, which experience a recovery, mainly in terms of weight , even between 4.5 and 12. 5 years of age [3].

Large multicenter studies reported an association between postnatal growth failure and poor neurodevelopmental outcome [4,5]. In particular, a small head size at 3 months of corrected age has been demonstrated to be associated to adverse neurodevelopmental outcome in early childhood [4,6].

Impaired neurodevelopmental outcome and postnatal growth failure can be interpreted as phenomena that probably share common nutritional and non-nutritional factors [5]. Although these phenomena cannot be completely corrected using only nutritional interventions, perinatal risk factors and neonatal complications alone do not fully explain the outcome [5]. In fact, the most recent evidence shows that careful nutritional interventions, in terms of protein intake and protein:energy ratio, can have a positive effect on neurodevelopmental outcome.

Particular attention must be paid to the quality of growth: organ growth and differentiation are influenced more by free-fat mass and linear growth than by weight or fat mass growth [7,8]. Moreover, recent evidence suggests a positive association between growth in terms of lean mass, but not fat mass, and better neurodevelopment in childhood [9].

Early catch–up growth (before 12-18 months of corrected age) in very preterm infants is associated with a lower percentage of body fat and is therefore likely to be beneficial in terms of neurological outcome [10] and not detrimental in terms of metabolic consequences in adulthood [11,12,13]. On the contrary, rapid weight growth happening later during childhood might negatively impact on adult health (diabetes, cardiovascular disease, hypertension).

**References**

1. da Silva Boguszewski MG, de Andre Cardoso-Demartini A. Growth and growth hormone therapy in short children born preterm hormone. European Journal of Endocrinology 2017; 176, R111–R122

2. Pierrat V, Marchand-Martin L, Guemas I, Matis J, Burguet A, Picaud JC, Fresson J, Alberge C, Marret S, Roze JC, Kaminski M, Larroque B, Ancel PY; the Epipage Study Group Height at 2 and 5 years of age in children born very preterm: the EPIPAGE study. Arch Dis Child Fetal Neonatal Ed 2011;96:F348–F354

3. Beukers F, Rotteveel J, van Weissenbruch MM, Ganzevoort W, van Goudoever JB, van Wassenaer-Leemhuis AG. Growth throughout childhood of children born growth restricted. Arch Dis Child 2017;102:735–741

4. Ehrenkranz RA, Dusick AM, Vohr BR, Wright LL, Wrage LA, Poole WK. Growth in the neonatal intensive care unit influences neurodevelopmental and growth outcomes of extremely low birth weight infants. Pediatrics 2006;117:1253–1261

5. Franz AR, Pohlandt F, Bode H, Mihatsch WA, Sander S, Kron M, et al: Intrauterine, early neonatal and postdischarge growth and neurodevelopmental outcome at 5.4 years in extremely preterm infants after intensive nutritional support. Pediatrics 2009; 123:e101–e109.

6. Neubauer V, Griesmaier E, Pehböck-Walser N, Pupp-Peglow U, Kiechl-Kohlendorfer U. Poor postnatal head growth in very preterm infants is associated with impaired neurodevelopment outcome. Acta Pædiatrica 2013;102:883–888

7. Ramel SE, Demerath EW, Gray HL, Younge N, Boys C, Georgieff MK. The Relationship of Poor Linear Growth Velocity with Neonatal Illness and Two-Year Neurodevelopment in Preterm Infants Neonatology 2012;102:19–24

8. Chien H-C , Chen C-H, Wang T-M, Hsu Y-C, Lin M-C. Neurodevelopmental outcomes of infants with very low birth weights are associated with the severity of their extra-uterine growth retardation. Pediatrics and Neonatology 2018;59:, 168e-175e

9. Scheurer JM, Zhang L, Plummer EA, Hultgren SA, Demerath EW, Ramel SE. Body Composition Changes from Infancy to 4 Years and Associations with Early Childhood Cognition in Preterm and Full-Term Children. Neonatology 2018;114:169–176.

10. Raaijmakers A, Jacobs L, Rayyan M, van Tienoven TP, Ortibus E, Levtchenko E, Staessen JA, Allegaert K. Catch–up growth in the first two years of life in Extremely Low Birth Weight (ELBW) infants is associated with lower body fat in young adolescence. PLOS ONE March 9, 2017, 1-15

11. Embleton ND, Korada M, Wood CL, Pearce MS, Swamy R, Cheetham TD. Catch-up growth and metabolic outcomes in adolescents born preterm Arch Dis Child 2016;101:1026–1031

12. Giannì ML, Roggero P, Piemontese P, Morlacchi L, Bracco B, Taroni F, Garavaglia E, Mosca F. Boys who are born preterm show a relative lack of fat-free mass at 5 years of age compared to their peers Acta Pædiatrica 2015;104:e119–e123

13. Lapillonne A, Griffin IJ. Feeding Preterm Infants Today for Later Metabolic and Cardiovascular Outcomes J Pediatr 2013;162:S7-16

## A10 Effects of infection severity on frail children: a comparison of epidemic seasons

### Renato Cutrera (renato.cutrera@opbg.net)

#### Pediatric Pulmonology and Sleep & Long-Term Ventilation Unit, Pediatric Intermediate Care Unit, Academic Pediatric Department, Pediatric Hospital “Bambino Gesù”, Rome, Italy

**Background:** respiratory syncytial virus (RSV) is the major pathogenic agent for lower respiratory infections in infants under one year of age [1,2] and represents the main cause of death in children with respiratory infections [2,5-8]. The major risk for severe RSV infection is prematurity [7-9], both in the extreme/preterm (< 28 weeks of gestational age (wGA) and in 28-36 wGA children [10]. The increased vulnerability of preterm is caused by the early interruption of pulmonary development and the physiologic immaturity of the immune system [11,12]. The presence of associated risk factors (low weight at birth, maternal smoking, age at the start of RSV epidemic season, male gender, absence/short breast-feeding, daycare attendance, presence of older siblings) cause a more severe infection [13-17] with an increased hospitalization risk. The only effective pharmacological prevention against RSV infection is Palivizumab, a humanized monoclonal antibody which binds the F protein on the virus surface, blocking fusion of the virus membrane with the target cell membrane [16]. Based on the first clinical studies and as recommended by the American Academy of Pediatrics (AAP), Palivizumab was authorized in 1998 by the Food and Drug Administration of the United States [18]. In 2014, a restrictive version of the AAP recommendations was published [19]: for healthy preterm, Palivizumab prophylaxis was recommended only for <29 wGA children <12 months old at the beginning of the RSV season. In Italy during the 2016-2017 season were adopted the AAP recommendations [20]. In consideration of the clinical evidence collected during the season the prophylaxis was allowed to preterm >29 wGA <6 months old at the beginning of the season [21].

**Conclusions**: the Italian clinical data confirm a high vulnerability for 29-36 wGA children and show how a decreased prophylaxis with Palivizumab in the 2016-2017 epidemic season was associated with a higher incidence of RSV bronchiolitis, and a greater respiratory function impairment. The 2016-2017 restriction caused an increase in number and length of hospitalizations and admissions to the Pediatric Intensive Care Units. An overall increase of the total number of RSV bronchiolitis during the 2016-2017 epidemic season has been observed, also in children born at term, suggesting that the decreased prophylaxis in preterm may have caused a wider infection diffusion also in children not considered at risk.

**References**

1. Nair H, Nokes DJ, Gessner BD, Dherani M, Madhi SA, Singleton RJ, et al. Global burden of acute lower respiratory infections due to respiratory syncytial virus in young children: a systematic review and meta-analysis. Lancet. 2010; 375: 1545–55.

2. Bont L, Checchia PA, Fauroux B, Figueras-Aloy J, Manzoni P, Paes B, et al. Defining the epidemiology and burden of severe respiratory syncytial virus infection among infants and children in western countries. Infect Dis Ther. 2016; 5: 271–98.

3. Bont L, Blanken M. Viral respiratory burden in moderate-to-late preterm infants. Early Hum Dev. 2013; 89 Suppl 1:S37-9

4. Hibbard JU, Wilkins I, Sun L, Gregory K, Haberman S, Hoffman M, et al. Respiratory morbidity in late preterm births. Consortium on safe labor. JAMA 2010; 304: 419-25.

5. Sanchez-Luna M, Elola FJ, Fernandez-Perez C, Bernal JL, Lopez-Pineda A. Trends in respiratory syncytial virus bronchiolitis hospitalizations in children less than 1 year: 2004–2012. Curr Med Res Opin. 2016; 32: 693–8.

6. Gupta P, Beam BW, Rettiganti M. Temporal trends of respiratory Syncytial virus-associated hospital and ICU admissions across the United States. Pediatr Crit Care Med. 2016;17:e343–51.

7. Hall CB, Weinberg GA, Iwane MK, Blumkin AK, Edwards KM, Staat MA, et al. The burden of respiratory syncytial virus infection in young children. N Engl J Med. 2009; 360: 588–98.

8. Lozano R, Naghavi M, Foreman K, Lim S, Shibuya K, Aboyans V, et al. Global and regional mortality from 235 causes of death for 20 age groups in 1990 and 2010: a systematic analysis for the global burden of disease study 2010. Lancet. 2012; 380: 2095–128.

9. Pulver LS, Denny JM, Silver RM, Young PC. Morbidity and discharge timing of late preterm newborns. Clin Pediatr Phila 2010; 49: 1061-7.

10. Quinn JA, Munoz FM, Gonik B, Frau L, Cutland C, Mallett-Moore T, Kissou A, Wittke F, Das M, Nunes T, Pye S, Watson W, Ramos AA, Cordero JF, Huang WT, Kochhar S, Buttery J; Brighton Collaboration Preterm Birth Working Group. Preterm birth: Case definition & guidelines for data collection, analysis, and presentation of immunisation safety data. Vaccine. 2016; 34: 6047-6056.

11. Langston C, Kida K, Reed M, Thurlbeck WM. Human lung growth in late gestation and in the neonate. Am Rev Respir Dis. 1984; 129: 607- 613.

12. Colin AA, McEvoy C, Castile RG. Respiratory morbidity and lung function in preterm infants of 32 to 36 weeks' gestational age. Pediatrics. 2010; 126: 115-28.

13. Corsello G, Di Carlo P, Salsa L, Gabriele B, Meli L, Bruno S, Titone L. Respiratory syncytial virus infection in a Sicilian pediatric population: risk factors, epidemiology, and severity. Allergy Asthma Proc. 2008; 29: 205-10.

14. Baraldi E, Lanari M, Manzoni P, Rossi GA, Vandini S, Rimini A, Romagnoli C, Colonna P, Biondi A, Biban P, Chiamenti G, Bernardini R, Picca M, Cappa M, Magazzù G, Catassi C, Urbino AF, Memo L, Donzelli G, Minetti C, Paravati F, Di Mauro G, Festini F, Esposito S, Corsello G. Inter-society consensus document on treatment and prevention of bronchiolitis in newborns and infants. Ital J Pediatr. 2014; 40: 65.

15. Lanari M, Prinelli F, Adorni F, Di Santo S, Vandini S, Silvestri M, Musicco M; Study Group of Italian Society of Neonatology. Risk factors for bronchiolitis hospitalization during the first year of life in a multicenter Italian birth cohort. Ital J Pediatr. 2015; 41: 40.

16. Blanken MO, Paes B, Anderson EJ, Lanari M, Sheridan-Pereira M, Buchan S, Fullarton JR, Grubb E, Notario G, Rodgers-Gray BS, Carbonell-Estrany X. Risk scoring tool to predict respiratory syncytial virus hospitalisation in premature infants. Pediatr Pulmonol. 2018; 53: 605-612.

17. Papoff P, Moretti C, Cangiano G, Bonci E, Roggini M, Pierangeli A, Scagnolari C, Antonelli G, Midulla F.; Incidence and predisposing factors for severe disease in previously healthy term infants experiencing their first episode of bronchiolitis. Acta Paediatr. 2011 Jul;100(7):e17-23. doi: 10.1111/j.1651-2227.2011.02181.x. Epub 2011 Mar 1

18. Subramanian KN, Weisman LE, Rhodes T, Ariagno R, Sánchez PJ, Steichen J, Givner LB, Jennings TL, Top FH Jr, Carlin D, Connor E. Safety, tolerance and pharmacokinetics of a humanized monoclonal antibody to respiratory syncytial virus in premature infants and infants with bronchopulmonary dysplasia. MEDI-493 Study Group. Pediatr Infect Dis J 1998; 17: 110–115.

19. American Academy of Pediatrics, Committee on Infectious Diseases and Committee on Fetus and Newborn. Prevention of respiratory syncytial virus infections: indications for the use of palivizumab and update on the use of RSV-IGIV. Pediatrics. 1998; 102:1211–1216.ex32

20. *AIFA.* Variazione del Piano Terapeutico Palivizumab. GU Serie Generale n.221 del 21-09-2016.

21. AIFA. Variazione del Piano Terapeutico Palivizumab. GU Serie Generale n.262 del 09-11-2017.

## A11 **Automated control** of FiO_2_ in respiratory assistance

### Carlo Dani (cdani@unifi.it)

#### Department of Neurosciences, Psychology, Drug Research and Child Health, University of Florence, Florence, Italy

**Background.** Oxygen-therapy in preterm infants is crucial for survival and at the same time a risk-factor for the development of severe complications such as bronchopulmonary dysplasia (BPD) and retinopathy of prematurity (ROP). To decide the optimal SpO_2_ target, but also to maintain this target during intensive neonatal care, is very important. Recently, systems allowing the automated control of FiO_2_ patient delivery to maintain SpO_2_ within a target range have been developed. These systems include a pulse oximeter, a non-invasive or invasive respiratory support (i.e. mechanical ventilator), and the algorithm that regulates the FiO_2_ adjustments in relationship to SpO_2_ changes. **Literature review.** Studies evaluating the effectiveness and clinical usefulness of these devices are heterogeneous for design and duration, gestational age and age at treatment, type of algorithm, and target SpO_2_.^1^ Thus, different clinical effects of systems for automated control of FiO_2_ in preterm infants may depend on many variables, such as population size, type of respiratory support, and chosen target range, but also reflect the effectiveness of the algorithm. **Main study results.** However, these studies demonstrate that patients spend more time within target SpO_2_ during automated control that during manual control, although this time greatly varies ranging from 40 to 91% for automated control and from 32 to 82% for manual control. ^1^ Moreover, it seems that automated control is more effective in reducing hyperoxia than hypoxia in comparison with manual control: this can be explained by the fact that closed loop systems can respond to episodes of oxygen desaturation with the automatic increase of FiO_2_ but are ineffective in preventing hypoxemia. ^1^ It would be interesting to evaluate if the width of the SpO_2_ target range may influence the system effectiveness: available data suggest that target range does not affect system effectiveness but the question is still debated. Similarly, the effectiveness of different systems for the automated control of FiO_2_ has not been evaluated since no studies used more than one device. No studies investigated whether automated control of FiO_2_ can affect outcome in preterm infants, and recently it has been questioned the actual possibility of these devices of beneficially influencing preterm infants’ outcome. **Conclusions.** Further clinical studies are required to assess the actual clinical effects of automated control of FiO_2_, determine most effective algorithms and, ultimately, to decide if these systems should become the standard of care.

**REFERENCE**

1. Dani C. Automated control of inspired oxygen (FiO2 ) in preterm infants: Literature review. Pediatr Pulmonol 2019;54:358-363.

## A12 Safety of newborns in the delivery room and during hospital stay

### Riccardo Davanzo^1^, Fabio Forte^2^, Carmela Giovanna Fabrizio^2^, Chiara Figliuolo^2^, Carmela Di Lucca^2^, Vincenza Targiani^2^, Angela Derosa^2^, Tiziana Faillace^2^

#### ^1^Scientific Direction, Institute for Maternal and Child Health IRCCS “Burlo Garofolo”, Trieste, Italy; ^2^Division of Pediatrics & Neonatology, Ospedale Madonna delle Grazie, Matera, Italy

##### **Correspondence:** Riccardo Davanzo (riccardo.davanzo@gmail.com)

**Background**

The historical displacement of childbirth from home to Maternity Hospital tremendously increased the safety of Reproductive Health. Yet, this clear improvement in perinatal health care has actually interfered with the naturality of mother and newborn infant relationship.

World Health Organization and UNICEF developed a structured Global Program called the Baby Friendly Hospital Initiative, launched in 1991, in order to restore maternal-infant bonding and exclusive breastfeeding.

Nevertheless, reorganization of a Maternity Hospital and implementation of appropriate hospital procedures and protocols is not an immediate nor an easy achievement.

Infact, while regaining a natural approach to childbirth, health professionals should simulataneously keep a highl level of safety for the newborn infant during hospital stay.

Therefore, newborns infants should be regularly monitored in the delivery room during skin to skin contact (SSC) and in the Rooming-in/Post-partum Ward.

Monitoring shoud address early identification of relatively common neonatal diseases such as respiratory distress or early onset sespis, but it is expected also to prevent sudden unexpected postnatal collapse (SUPC), particularly in the first 2 hours of life.

Certainly, SSC may ease the infant’s transition to extra uterine life and helps to regulate the infant’s body temperature and nursing behavior. Nevertheless SSC has also been associated to sudden unexpected cardio-respiratory events of the newborn infant, who deserves careful monitoring.

**Results**

A dedicated check list has been developed in our hospital to increase newborn safety in the first 2 hours of life [1, 2] (Table 1).

Moreover, according to current recommendations from the Italian Society of Pediatrics (SIP) and the Italian Society of Neonatology (SIN)(1), midwives/nurses should strictly observe healthy newborn infants during the transition period (first 6-12 h after birth). At least 2 observations should be made between 3 and 12 h after birth. After the transition period or after the first 12 h of life, mother-baby dyads should be observed at least once every 8 h until hospital discharge.

Lastly, bed-sharing should be discouraged as it has been proven to be an harmful practice (1).

**Conclusion**

Far from being proven effective to prevent SUPC, the routine use of a check-list for neonatal monitoring still represents a tool for a best practice approach.

**References**

1. Piumelli R, Davanzo R, Nassi N et al. ALTE: Italian Guidelines. Italian Journal of Pediatrics 2017: 43: 111

2. Davanzo R, De Cunto A, Paviotti G et al. Making the first days of life safer: preventing sudden unexpected postnatal collapse while promoting breastfeeding. J Hum Lact. 2015 Feb;31(1):47-52. doi: 10.1177/0890334414554927. Epub 2014 Oct 22.

**Table 1 (abstract A12).** Check-list for newborn infants in the first 2 h of life, particularly during the skin to skin contact

FAMILY NAME AND NAME:		Times after birth				
DATE OF BIRTH:	HOUR OF BIRTH: …./….					
PARAMETERS TO BE ASSESSED OR EVENTS TO BE RECORDED		10 min*	30 min	60 min	90 min	120 min
Correct position of the newborn with mouth and nose visible and unobstructed (the assessment of this parameter does not require separation of the newborn from the mother’s chest or interruption of a possible first feed): YES/NO						
Pink colour (skin and/or mucous membranes): YES/NO						
Normal breathing (no retractions or grunting or flaring of the nostrils): YES/NO						
Normal respiratory rate (30-60 breaths/min): YES/NO						
Normal S_p,O2_: > 90% (if deemed necessary): YES/NO						
Skin temperature at 60 and 120 minutes after birth Normal reference values: 36.5-37.5 °C						
Mother never left alone with the newborn: YES/NO						
First breastfeeding attempt (time)						
Comments						
Signature (midwife/nurse/doctor)						

## A13 Metabolomics for early detection of preterm newborns with early onset sepsis

### Enrica Donadel^1^, Veronica Mardegan^2^, Matteo Stocchero^3^, Giuseppe Giordano^3^, Elena Priante^2^, Paola Pirillo^3^, Margherita Magnani^1^, Marta Meneghelli^1^, Sara Rossin^1^, Sabrina Salvadori^2^, Giovanna Verlato^2^, Eugenio Baraldi^2,3^

#### ^1^Specialization School in Pediatrics, Health Department of Woman and Child, University of Padua, Padua, Italy; ^2^Neonatal Intensive Care Unit, Health Department of Woman and Child, University of Padua, Padua, Italy; ^3^Pediatric Research Institute Città della Speranza, Padua, Italy

##### **Correspondence:** Enrica Donadel (enrica.donadel@gmail.com)

**Background**

Neonatal sepsis is a complex infection-induced systemic inflammatory response syndrome and it is a main cause of mortality and neurologic sequelae in newborns.

An early and accurate detection of sepsis is mandatory in neonates, since the clinical course of the disease can be fulminant. The gold standard for diagnosis of neonatal sepsis is blood culture, but results are not quickly available and false negative results are not rare. To date, there is still no reliable biochemical marker of neonatal sepsis.

Metabolomics represents the analysis and interpretation of global metabolic data of a complex biological system. In sepsis, metabolomics is a very promising field of research [1].

The aim of the study is to compare the metabolic profile of urine collected within 72 hours of birth between preterm neonates affected by early onset sepsis (EOS) and healthy preterm infants, looking for a specific metabolic profile able to early detect preterm newborns prone to develop EOS.

**Materials and methods**

Each preterm newborn admitted to the Neonatal Intensive Care Unit from December 2015 to November 2017 was recruited. Infants who developed a septic episode within 72 hours of birth were enrolled as cases. Infants who did not developed sepsis were enrolled as controls. For each subject, urine samples were collected within 72 hours of birth. The urine samples underwent untargeted metabolomic analysis using mass spectrometry combined with ultra-performance liquid chromatography. The data obtained were analyzed using multivariate and univariate statistical data analysis tools.

**Results**

One-hundred and twenty-three subjects were enrolled in the study. Seventeenth neonates with EOS were identified as cases (mean gestational age 210 days + 18 days SD, mean birth weight 1339 g + 394 g SD). Seventeenth gestational age-matched newborns were enrolled as controls (mean gestational age 214 days + 16 days SD, mean birth weight 1298 g + 333 g SD). Metabolic profiles of urine samples collected within 24 hours of birth revealed a statistically significant separation between septic and non-septic neonates. Metabolic pathways predominantly perturbed in septic newborns were taurine/hypotaurine and cysteine/methionine metabolism, phenylalanine/tyrosine/tryptophan biosynthesis, nitrogen metabolism.

**Conclusions**

At the onset of infection, neonates with EOS show a distinctive urine metabolic profile that allows their early identification by non-septic newborns. Targeted analysis of annotated metabolites belonging to metabolic pathways predominantly perturbed in septic newborns could shed light on the complex pathophysiological machanisms involved with sepsis and potentially provide new potential biomarkers for early diagnosis of sepsis.

**Ethics Approval**

The study was approved by the local Ethics Committee.

**Reference**

1. Fanos V, Caboni P, Corsello G. Urinary ^1^H-NMR and GC-MS metabolomics predicts early and late onset neonatal sepsis. Early Hum Dev. 2014; 90S1:S78-83.

## A14 The enhancement of the nursing role in the Single Family Room in NICU

### Fasulo Martina, Daniela Motta, Arienti Benedetta, Ludrini Laura, Valentina Galbusera, Mandelli Carlotta, Raso Matteo, Sala Patrizia

#### Neonatal Intensive Care Unit, MBBM Foundation, San Gerardo Hospital Monza, Italy

##### **Correspondence:** Fasulo Martina (marty.fasulo@gmail.com)

**Background.** The subject of the following paper is to investigate the nursing role within the Neonatal Intensive Care Unit of Monza, a department structured according to the project of the Single Family Rooms. It has always been the desire and objective of the nursing and medical staff to focus care on the child and his family.

For this reason, in 2009 the opening of the open space department to parents was experienced for 24 hours. The organisational structure limited the time that parents could spend in the ward and therefore their involvement in the childcare plan. Later, in 2017, the SFR department was opened and this gave the possibility to implement the participation of parents ensuring greater comfort and privacy.

It is in this specific aspect that the enhancement of the nursing role emerges: from a Family Centered Care model, we are gradually moving to a Family Integrated Care model. This new model of neonatal care supports parents as primary caregivers and partners of the clinical group^1,2,3^; in addition to a physical involvement of the family, the most interesting aspect remains the important change in the cultural and relational level of the staff. The biggest challenge is in fact the constant adherence and commitment of the nursing group that from main caregiver is transformed into educator and supporter integrating and accepting parent as active members of the infant's team^1,2,3,4,5,6^.

**Conclusion.** Being the first reality in Italy to introduce this welfare model, the entire staff was also faced with a series of organizational and relational aspects of primary importance for the success of the experiment. The awareness of being a reference model for other structures and the determination to achieve the goal in an optimal way, have pushed the staff to continuously search for effective strategies to overcome any obstacle that could compromise the final result.

**References**

1. Craig JW, Glick C, Phillips R, Hall SL, Smith J, Browne J.: Recommendations for involving the family in developmental care of the NICU baby. J Perinatol. 2015 Dec;35 Suppl 1:S5-8.

2. Stevens DC, Helseth CC, Khan MA, Munson DP, Smith TJ. Neonatal intensive care nursery staff perceive enhanced workplace quality with the single-family room design. J Perinatol. 2010 May;30(5):352-8

3. Watson J, DeLand M, Gibbins S, MacMillan York E, Robson K. Improvements in staff quality of work life and family satisfaction following the move to single-family room NICU design. Adv Neonatal Care. 2014 Apr;14(2):129-36.

4. Patel, Ainsley Ballantyne, Gillian Bowker, Jack Weightman, Sarah Weightman, Family Integrated Care: changing the culture in the neonatal unit. Neil for the Helping Us Grow Group (HUGG)Patel N, et al. Arch Dis Child May 2018 Vol 103 No 5

5. Karel O’Brien, Kate Robson, Marianne Bracht, Melinda Cruz, Kei Lui, Ruben Alvaro, Orlando da Silva, Luis Monterrosa, Michael Narvey, Eugene Ng, Amuchou Soraisham, Xiang Y Ye, Lucia Mirea, William Tarnow-Mordi, Shoo K Lee, for the FICare Study Group and FICare Parent Advisory Board, Effectiveness of Family Integrated Care in neonatal intensive care units on infant and parent outcomes: a multicentre, multinational, cluster-randomised controlled trial, Published online February 7, 2018 http://dx.doi.org/10.1016/S2352-4642(18)30039-7

6. Amy Young, Liz McKechnie, Catherine M Harrison, Family integrated care: what’s all the fuss about?, Arch Dis Child Fetal Neonatal Ed March 2019 Vol 104 No 2

## A15 Patent ductus arteriosus: diagnosis and treatment news

### Benjamim Ficial, Elena Bonafiglia, Francesca Bissolo, Carlo Alberto Forcellini, Irene Sibona, Mariela Ventola, Paolo Biban

#### Neonatal Intensive Care Unit, Azienda Ospedaliera Universitaria Integrata di Verona, Verona, Italy

##### **Correspondence:** Benjamim Ficial (benjamim.ficial@gmail.com)

**Background**

Despite decades of research, there is still no consensus on the optimal management of patent ductus arteriosus (PDA) in preterm infants. PDA closure may be delayed, resulting in pulmonary overcirculation and systemic hypoperfusion. These haemodynamic consequences are determined by the magnitude of the ductal shunt together with the cardiac and pulmonary responses to that shunt.

**Materials and methods**

Literature review.

**Results**

A comprehensive echocardiographic assessment is currently recommended to identify the neonates exposed to a moderate/large ductal shunt. Recent data showed that early screening echocardiography to target treatment may improve outcome. The correct timing for the screening seems to be the second day of life, when there is a statistically significant difference in echocardiographic parameters between babies with a small and large shunt. Recently a consensus statement has been released aiming to standardize the assessment of haemodynamic significance of PDA [1].

Correctly identifying neonates with moderate/large ductal shunt plays a pivotal role: in fact, a consistent number of randomized controlled trials (RCTs) showed that early non selective pharmacological treatment has no effect on death, bronchopulmonary dysplasia (BPD), necrotizing enterocolitis (NEC) and neurosensory impairment. In the last ten years, this evidence paved the way to a more conservative approach, that led to lower rates of medical and surgical closure.

However, these RCTs have two main confounding factors: first, the magnitude of the ductal shunt was not assessed; second, the control group was not exposed to a prolonged ductal shunt, due to the high rate of spontaneous closure or rescue treatment.

There is emerging evidence from quality improvement trials that prolonged exposure to high/moderate ductal shunt for the first 7–10 days after birth is associated with BPD and that pharmacological treatment might help [2].

Current pharmacological treatment includes ibuprofen, indomethacin and paracetamol. Recent data in preterm infants treated beyond the first week of life showed a similar efficacy for indomethacin and ibuprofen, with indomethacin causing a greater constriction, whilst paracetamol had a reduced effect [3].

Recently, transcatheter PDA closure, performed at the bedside with an antegrade femoral venous approach, is emerging as a viable option even in preterm neonates. Although it looks promising, feasibility of this procedure within the first 10 days of life, the “critical window” for BPD development, and comparison with other treatment modalities should undergo further assessment [4].

**Conclusions**

Further studies are urgently needed to guide appropriate and effective treatment in babies with PDA.

**References**

1. van Laere D, van Overmeire B, Gupta S, El Khuffash A, Savoia M, McNamara PJ, et al. European Special Interest Group “Neonatologist Performed Echocardiography” (NPE). Application of NPE in the assessment of a patent ductus arteriosus. Pediatr Res 2018;84(Suppl 1):46-56.

2. Clyman RI. Patent ductus arteriosus, its treatments, and the risks of pulmonary morbidity. Semin Perinatol 2018;42(4):235-242.

3. Liebowitz M, Kaempf J, Erdeve O, Bulbul A, Håkansson S, Lindqvist J, et al. Comparative effectiveness of drugs used to constrict the patent ductus arteriosus: a secondary analysis of the PDA-TOLERATE trial (NCT01958320). J Perinatol 2019;39:599–607.

4. Almeida-Jones M, Tang NY, Reddy A, Zahn E. Overview of transcatheter patent ductus arteriosus closure in preterm infants. Congenit Heart Dis 2019;14:60–64.

## A16 Surfactant administration with less invasive technique: scientific evidence, clinical practice and future perspectives

### Francesca Castoldi, Gianluca Lista

#### Department of Pediatrics-NICU-Ospedale dei Bambini“V.Buzzi” ASST-FBF-Sacco, Milan, Italy

##### **Correspondence:** Gianluca Lista (gianluca.lista@asst-fbf-sacco.it)

Premature infants with Respiratory Distress Syndrome (RDS) are usually managed with mechanical ventilation plus exogenous surfactant administration, or INSURE (INtubation-SURfactant administration-Extubation) technique in course of non invasive respiratory support (e.g. nasal CPAP). In order to try to avoid tracheal intubation and some risks related to mechanical ventilation (e.g. baro-volutrauma), surfactant administration with less invasive technique (e.g. LISA) is becoming very popular since its positive correlation with improved pulmonary outcomes and/or increased survival without major complications [1-2]. These findings are limited by the overall low quality of evidence deriving from the available studies. In fact many trials are not blinded, the surfactant dose and the catheters are not always comparable between the studies, the comparison of outcomes between LISA and INSURE or traditional modality to deliver surfactant has been obtained by restrospective or observational studies, etc. A recent European survey [3] reported that also in clinical practice, there is a wide variation in team views on patients selection, in time used for surfactant delivery in course of LISA, in policies concerning premedications, sedation and devices [3]. The extremely low for gestational age (ELGA) infants, that are generally affected by RDS, are a high risk population for adverse long-term outcomes (e.g. BPD and neurological impairment). In these babies with a gestational age less than 26 wks, safety and efficacy of LISA need to be verified much better with future and well-powered RCTs. Moreover, the best modality of non invasive respiratory support (e.g nasal CPAP or NIPPV, NSIPPV, etc) has not been well investigated until now. Premedications and sedation (both pharmacological and not) are now under investigation by numerous trials ongoing in the world and probably in the future we will have more clear indication for their use in course of LISA technique. Probably because of these uncertainties, some neonatologists still prefer to use INSURE instead of LISA to give surfactant in preterm infants in non invasive respiratory support for RDS. Animal data on pulmonary surfactant distribution, oxygenation and lung mechanics changes in course of LISA within specific strategies (e.g. lung recruitment before surfactant, modality of non invasive respiratory support, etc) and well-powered RCTs may probably help neonatologists in better understanding the real efficacy of LISA in RDS management and its effect on long term respiratory and neurological outcomes.

**References**

1. Isayama T, Iwami H, McDonald S, Beyene J. Association of noninvasive ventilation strategies with mortality and bronchopulmonary dysplasia among preterm infants: a systematic review and meta-analysis. Jama 2016; Aug 9; 316(6):611-24

2. Hartel C, Paul P, Hanke K, Humberg A, Kribs A, Mehler K et al. Less invasive surfactant administration and complications of preterm birth. Sci Rep 2018;May 29; 8(1):8333

3. Klotz D, Porcaro V, Fleck T, Fuchs H. European perspective on less invasive surfactant administration-a survey. Eur J Pediatr 2017;Feb 176(2):147-154

## A17 Nursing training and competence in neonatology

### Maria N. Login^1^, Giulia Donati^2^, Federica Tomassini^3^, Maria L. Rega^4^

#### ^1^Neonatal Intensive Care Unit, Agostino Gemelli University Polyclinic, Rome, 00168, Italy; ^2^Agostino Gemelli University Polyclinic, Rome, 00168, Italy; ^3^Agostino Gemelli University Polyclinic, Rome, 00168, Italy; ^4^School of Nursing, Catholic University of the Sacred Heart, Rome, 00168, Italy

##### **Correspondence:** Maria N. Login (marianicoleta.login@policlinicogemelli.it)

**Background**

Neonatology is a distinct specialty with the need for specific skills and knowledge areas. In addition the acquisition of these skills and knowledge is essential for the delivery of safe and effective care to the neonate. To ensure the quality of care and provide the safety of patients, evaluating the clinical competence of nurses seems necessary. However, analyzing the scientific literature regarding the nursing advanced competence in neonatology, very few studies specify and define postgraduate university education required of professionals in the neonatology. There are several studies that support the importance of advanced expertise and specialization of the neonatal intensive care nurse and suggest they improve patient outcomes.

**Materials and methods**

The first stage of the study focused on a literature review regarding postgraduate university education of nurses in the neonatology. In a second stage a questionnaire was administered among neonatal intensive care unit (NICU) nurses working in a university hospital in Rome, Italy, to assess the perceptions of nurses regarding standardized training courses.

**Results**

Since nursing care for premature newborn is not included in the academic training, nurses do not receive specific training courses before working formally in NICU. The unique aspects and the complexity of the NICU, in addition to the vulnerability of the neonatal population require standardized training courses to improve and enhance the clinical competence of nurses in performing their professional tasks and roles in a safe way and with good quality.

**Conclusions**

Most of the nursing professionals consulted consider that their postgraduate academic training is not appropriate to their workplace. Studying nurses' training demands, the data obtained may help in designing education strategies which should be established and their effectiveness evaluated.

**Acknowledgements**

We would like to thank Professor Giovanni Vento for the valuable contribution and constructive collaboration, we would like also to thank all of our colleagues for the support and participation.

**References**

Aari RL, Tarja S, Helena LK. Competence in intensive and critical care nursing: a literature review. Intensive Crit Care Nurs. 2008 ; 24:78-89

Alfieri E, Alebbi A, Bedini MG, Boni L, Foà C. Mapping the nursing competences in neonatology: a qualitative research. Acta Biomed. 2017 ; 88:51-58.

Cavaliere TA, Sansoucie DA. The use of family nurse practitioners and pediatric nurse practitioners as providers of neonatal intensive care: Safe practice or risky business? Newborn Infant Nurs Rev. 2001 ;1:142–7.

Garside JR, Nhemachena JZ. A concept analysis of competence and its transition in nursing. Nurse Educ Today. 2013;33:541-5.

Kamel SSA, Fakhry SF, Ibrahim RM. Self-assessment competency tool for nurses working in critical care units: development and psychometric evaluation. Life Science Journal. 2011;8:156–64.

Phibbs CS, Baker LC, Caughey AB, Danielsen B, Schmitt SK, Phibbs RH. Level and volume of neonatal intensive care and mortality in very-low-birth-weight infants. N Engl J Med 2007;356:2165–75.

Smith SA. Nurse competence: a concept analysis. Int J Nurs Knowl. 2012; 23:172-82.

Windsor C, Douglas C, Harvey T. Nursing and competencies — a natural fit: the politics of skill /competency formation in nursing. Nurs Inq 2012; 19:213–22.

Yanhua C, Watson R. A review of clinical competence assessment in nursing. Nurse Educ Today. 2011;31:832-6.

## A18 Neurocognitive long-term follow-up of ex-premature children and correlation with their functional MRI

### Laura Lorioli^1^, Flavia Leone^1^, Pasquale Anthony Della Rosa^3^, Matteo Canini^**3**^, Cristina Baldoli^**3**^, Roberta Longaretti^1^, Paola Scifo^4^, Rosanna Rovelli^1^**,** Andrea Falini^3^**,** Graziano Barera^1^, Antonella Poloniato^1^

#### ^1^Department of Neonatology, IRCCS San Raffaele Scientific Institute, Milano, Italy; ^2^Department of Neuroradiology, IRCCS San Raffaele Scientific Institute, Milano, Italy; ^3^Department of Nuclear Medicine, IRCCS San Raffaele Scientific Institute, Milano, Italy

##### **Correspondence:** Antonella Poloniato (poloniato.antonella@hsr.it)

Background

Despite the remarkable improvement in survival, preterm birth represents a major public health problem. Preterm survivors could experience neurocognitive long-term complications and neurodevelopmental disabilities represent one of the major concerns in this population, even in the absence of detectable brain lesions. Prematurity may likely spill into neurodevelopmental hindering throughout life by translating into visual and hearing disorders, motor deficits and cognitive impairments. Abnormal connectivity within key functional networks may underlie the higher degree of language, communication and attention problems found in preterm children^1^.

The aim of the study was: 1) to compare neurocognitive outcomes in the language and attention domain between a group of children born preterm and age-matched at-term born counterparts, 2) to correlate the functional MRI (fMRI) BOLD estimates acquired for the same group of preterm children at 39 gestational week (GW) during a language task, with their neurodevelopmental outcome in the school-age period, focusing on language and attention, in order to explore the relationship between neonatal preterm brain function and potential neurocognitive frailties arising at later developmental stages.

Materials and Methods

We enrolled 9 children born preterm (GW 29-32), aged between 10-12 years. All preterm children previously underwent a neonatal auditory fMRI after reaching at term equivalent age^2^. Anthropometric evaluation, structural and functional fMRI were performed and a neuropsychological battery of 18 NEPSI-II subtests (assessing Attention and Executive Functioning, Language, Memory and Learning, Visuospatial processing, Social Perception and Sensorimotor Functioning) was administered to all preterms at school-age and to a group of 8 age-matched at-term born children.

The study protocol was approved by San Raffaele Hospital Ethical Committee.

Results

A two-sample t-test between preterm children and age-matched control counterparts revealed a significant difference in the attention subdomain of NEPSI-II, specifically for the inhibition subtest (p=0.02; preterm mean score 8.78; control mean score: 11.38). Furthermore, BOLD neonatal fMRI parameter estimates extracted from several temporal, occipital and parietal regions were found to be significantly associated with neurocognitive outcome for the inhibitory subtest and for other subtests in both the attention and language subdomains.

Conclusions

fMRI neonatal measurements of brain function seem to be already associated somehow to neurodevelopmental outcome in apparently healthy ex-preterm children at school-age. Prompt identification of functional neonatal hallmarks of cognitive development allowing an early diagnosis of potential neurocognitive deficits that otherwise would manifest only during the school age could foster early therapeutic intervention, being of great help in starting dedicated rehabilitative programs in early childhood.

References

1. Rogers CE, Lean RE, Wheelock MD, Smyser CD. Aberrant structural and functional connectivity and neurodevelopmental impairment in preterm children. J Neurodev Disord. 2018 Dec 13;10(1):38. doi: 10.1186/s11689-018-9253-x.

2. Baldoli C, Scola E, Della Rosa PA, Pontesilli S, Longaretti R, Poloniato A, Scotti R, Blasi V, Cirillo S, Iadanza A, Rovelli R, Barera G, Scifo P. Maturation of preterm newborn brains: a fMRI-DTI study of auditory processing of linguistic stimuli and white matter development. Brain Struct Funct. 2015 Nov;220(6):3733-51. doi: 10.1007/s00429-014-0887-5. Epub 2014 Sep 23. PMID: 25244942

## A19 Extrauterine growth restriction (EUGR) in infants born before 30 weeks of gestational age: comparison of diverse definitions in association with neurodevelopmental and auxological outcomes at 24 months of corrected age

### Giulia Maiocco^1^, Francesco Cresi^1^, Elena Spada^1^, Paola Di Nicola^1^, Chiara Peila^1^, Vito Andrea Dell’Anna^1^, Maurizio Turin^1^, Stefano Sottemano^1^, Elena Maggiora^2^, Enrico Bertino^1^, Alessandra Coscia^1^

#### ^1^Neonatal Unit of the University, City of Health and Science of Turin, Turin, Italy; ^2^Neonatal Intensive Care Unit of C. Arrigo Pediatric Hospital, Alessandria, Italy

##### **Correspondence:** Giulia Maiocco (giulia.maiocco@edu.unito.it)

**Background**

Very preterm infants (GA <32) frequently present EUGR; a number of different definitions could be used in describing EUGR, namely cross-sectional EUGR as a measurement < than a defined percentile/SDS at 36 or 40 weeks of postmenstrual age (PMA), or at discharge; longitudinal EUGR as a ΔSDS < –1 or –2 between measurements at birth and at 36 or 40 weeks of PMA, or at discharge.

**Objective**

Highlight the association between EUGR and neurodevelopmental and auxological long-term outcomes for infants <30 weeks of GA. Comparison of predictive values of different EUGR definitions.

**Methods**

A total of 211 infants <30 weeks assisted at the NICU at University of Turin between 2006 and 2016 were enrolled. All infants had been followed-up until 24 months of corrected age (CA).

EUGR was calculated both for weight and head circumference (HC). Cross-sectional EUGR was defined as a measurement <10th percentile at 36 or 40 (± 1) weeks of PMA. Longitudinal EUGR was defined at different time intervals from those previously described in Literature, i.e. from 14 or 21 (± 3) days of life to 36 or 40 (± 1) weeks of PMA. Thus, the 2-3-week physiological period of weight loss is excluded, and only postnatal measurements are used (SDS values were assessed according to the Intergrowth-21^st^ longitudinal Charts for Postnatal Growth of Preterm Infants).

Neurodevelopmental outcomes were divided into major and minor impairments; auxological outcomes were assessed as continuous variables in SDS of weight or height, according to the WHO Child Growth Standards. A multivariable analysis was performed for major neonatal comorbidities, gestational age, and birth weight.

**Results**

Minor impairments are significantly associated with longitudinal definition of EUGR between 14 days of life and 36 weeks of PMA for HC while borderline significance is found for weight. Conversely, cross-sectional definitions of EUGR are not associated with neurodevelopmental outcomes. Auxological outcomes at 24 months of CA are predominantly influenced by birth weight rather than by EUGR.

**Conclusions**

Longitudinal definition of growth restriction would appear to be more appropriate than the cross-sectional definition since it is predictive of long-term neurodevelopmental outcomes. The association is of particular clinical interest for HC. This study may provide useful information for a new definition of EUGR, which is calculated entirely longitudinally using specific longitudinal anthropometric charts for preterms, starting from 14-days of age to 36 weeks of PMA.

## A20 Family-centered care in Italian NICUs: a long way home

### Giuseppe Paterlini^1^, Claudia Artese^2^

#### ^1^UO Neonatologia e TIN, Fondazione MBBM, Ospedale S. Gerardo, Monza, Italy; ^2^Dipartimento Professioni Sanitarie, SOD di Neonatologia e TIN AOU Careggi, Florence, Italy

##### **Correspondence:** Giuseppe Paterlini (giuseppe.paterlini@unimib.it)

**Background**

Preterm birth is a risk factor for the psychomotor, behavioural and affective-relational development of the child. The incidence of overall morbidity and the high risk of developmental impairment require that the care of these infants is increasingly focused on the prevention of neurological outcomes, aimed at encouraging personalized, family-centred care to improve the quality of life of these children [1].

Parental engagement plays a fundamental role in enhancing therapeutic and care processes.

The Working Group on Neonatal Developmental Care of the Italian Society of Neonatology carried out several studies to assess the level of attention regarding the issues of family-centered care in Italy.

**Results**

A survey published in 2009 [2] revealed that mothers are allowed unrestricted access in 29% NICUs, individualized developmental care procedures and breast milk feeding at discharge were uncommon and kangaroo care (KC) was performed in 67% of NICUs.

In 2012 a European study [3] reported encouraging parental participation in the care of the babies only in 80% of the Italian NICUs, furthermore many units applied restrictions regarding its frequency, method, and clinical conditions that would prevent KC practice.

In 2017 we proposed a new questionnaire to assess attitudes towards the parents, KC practice and breastfeeding support. We recruited 86/107 NICUs admitting infants ≤ 32 weeks of gestation.

An unrestricted entry was declared by 62% of the units.

The KC is offered in 94% of the NICUs but only in 38% of them without time restrictions and with greater limitations for the fathers. The average duration of one session is about 1h and 40m, the daily length is about 2h 40m. The average KC session length was significantly (p<0,02) greater in unrestricted access NICUs, and it wasn’t related to the number of nurses (NICU bed/nurses).

In 57% of the centers KC stops at the discharge of the newborn. The KC is not reported in the medical record in 40% of the NICUs, only in 42.7% of the cases there are written KC protocols.

**Conclusions**

Despite parental involvement is widely accepted, specific policies and practices to encourage it are still very poor and suffer from established habits.

KC is substantially widespread in surveyed NICUs, its application is still inadequate in terms of time and duration, compared to WHO recommendations [4] and inferior to other European countries.

It’s paramount to continue to encourage and support actions aimed to favour the active involvement of parents and to promote policies of openness of NICUs entry.

Acknowledgements

On the behalf of Gruppo di Studio Care Neonatale - SIN

References

1. Potharst ES, van Wassenaer AG, et al. High incidence of multi-domain disabilities in very preterm children at five years of age. J Pediatr 2011;159(1):79-85.

2. de Vonderweid U, Leonessa M. Family centered neonatal care. Early Hum Dev. 2009; 85: S37–S38.

3. Pallás-Alonso CR, Losacco V, Maraschini A, Greisen G, Pierrat V, Warren I, Haumont D, Westrup B, Smit BJ, Sizun J, Cuttini M, European Science Foundation Network. Parental involvement and kangaroo care in European neonatal intensive care units: a policy survey in eight countries. Pediatr Crit Care Med. 2012;13(5):568-77.

4. WHO. Kangaroo Mother Care: a practical guide. Geneva: WHO, 2003. Available at: http://apps.who.int/iris/bitstream/handle/10665/42587/9241590351.pdf;jsessionid=4442D61D929FA47559F37F81977EBB04?sequence=1, last access: July 2018.

## A21 Neuroimaging and antiviral therapy in citomegalovirus infection: Gaslini experience as a pilot phase to launch a multicenter study

### Giulia Polleri^1*^, Alessandro Parodi^2^, Chiara Andreato^1^, Alessandra Maggioni^1^, Elisabetta Godano^1^, Federica Mongelli^1^, Noemi Brolatti^1^, Giorgia Rossi^1^, Mariya Malova^2^, Andrea Rossi^3^, Domenico Tortora^3^, Emilio M Cristina^4^, Elio Castagnola^4^, Luca A Ramenghi^12^

#### ^1^University of Genoa, Genoa, Italy; ^2^Neonatal Intensive Care Unit, IRCCS Istituto G. Gaslini, Genoa, Italy; ^3^Neuroradiology Unit, IRCCS Istituto G. Gaslini, Genoa, Italy; ^4^Infectious Diseases Department, IRCCS Istituto Giannina Gaslini, Genoa, Italy

##### **Correspondence:** Giulia Polleri (pollerigiulia@gmail.com)

**Background**

The management of newborns affected by congenital CMV infection (cCMV) is based on rigourous diagnostic criteria prior to administration of ganciclovir/valganciclovir.

The definition of "symptomatic" patient remains problematic even when neuroimaging criteria are used.

Treating neonates with mild abnormalities on MRI is challenging as these patiens (i.e. T2 signal prolongation in the white matter suggesting inflammation) may develop long term neurological sequelae. Medical treatment is more often started with overt signs of severe brain damage (i.e. polymicrogyria, temporal cysts, severe ventricualr dilatation).

The objectives of this study are to 1) evaluate the frequency of patients with symptomatic cCMV; 2) identify the frequency of mild neuroradiological alterations (germinolytic cysts and / or T2 hyperintensity of white matter on brain MRI as the only sign of cCMV; 3) ascertain the treatment modality chosen.

**Materials and methods**

We performed a single-center retrospective study where we included all full-term infants with cCMV born between January 2012 and April 2019 admitted to our Institute.

Antiviral therapy has been proposed to parents of newborns with at least 1 clinical /neuroradiological sign of CNS involvement (deafness, microcephaly, neuroradiological changes on MRI, chorioretinitis, altered neurological examination) or with at least 2 signs of extracerebral involvement (petechiae, thrombocytopenia, hepatitis, etc.) [1,2]

**Results**

We included 39 full-term newborns with cCMV, 25/39 (64.1%) showed signs of symptomatic disease with indication for antiviral treatment. Severe brain abnormalities were present in 8/25 of symptomatic newborns (32%), while in the remaining 17/25 cases (68%) only mild abnormaliteis were detectable. In this group, they were the only sign of cCMV in nine (9/17) patients. Antiviral treatment was accepted by parents in 2 of the aforementioned 9 cases. Overall, antiviral therapy was performed in 8/25 symptomatic cases (32%).

**Conclusions**

We identified mild neuroradiological changes in 23% of newborns with cCMV as the only sign of disease; within this subgroup, a minority of patients received antiviral therapy.

We have undertaken an international multicenter study in order to evaluate the prognostic significance of these mild and isolated neuroradiological alterations, which are not included among the inclusion criteria of the two randomized contolled trials on which the current recommendations are based. [3,4]

1. Luck SE, et al. Pediatr Infect Dis J 2017;36(12):1205-1213.

2. Rawlinson WD, et al. Lancet Infect Dis 2017;17(6):e177-e188.

3. Kimberlin DW, et al. J Pediatr 2003;143(1):16-25.

4. Kimberlin DW, et al. N Engl J Med 2015;372(10):933-43.

## A22 Central venous access in neonates: the soft revolution

### N. Pozzi^1^, G. Barone^2^, A. Capasso^3^

#### ^1^Neonatal Intensive Care Unit, Maternal and Child Health Department, San Pio Hospital, Benevento, Italy; ^2^Neonatal Intensive Care Unit, Maternal and Child Health Department, Infermi Hospital, Rimini, Italy; ^3^Neonatal Intensive Care Unit, Department of Translational Medical Science, Federico II University, Naples, Italy

##### **Correspondence:** N. Pozzi (nicolapozzi71@gmail.com)

**Background**

The placement of a central venous catheter (CVC) is the most common procedure in Neonatal Intensive Care Units (NICUs). CVCs are mainly used to provide parenteral nutrition but they are also crucial to infuse inotropes or drugs which cannot be administered over a peripheral route. In the last decade, the world of vascular access is completely changed in the adult and pediatric fields due to the recent technological and methodological advancements.

**Objectives**

To define this slow but inexorable revolution/innovation that is spreading in the world of vascular access in the neonatal field.

**Methods**

To review the data from the Pediatric branch of the Association for Vascular Access (PEDIVAN) focusing on three main aspects: to define the role of ultrasound (US)-guided CVCs in term and premature infants compared to the traditional epicutaneous-caval catheters (ECC); to define the cost-effectiveness of the Near-Infrared Technology (NIR) in easing the insertion of ECCs; to implement the use of new technologies in NICU.

**Results**

The potential advantages of US-guided CVCs are mainly related to the use of last generation polyurethane catheters which are suitable for blood sampling, high flow rate infusion of fluids and hemodynamic monitoring [1]. The exact role of such catheters versus ECC in neonates is still to be defined. It’s likely that stable newborns who just need parenteral nutrition might benefit more from ECC while unstable and sick neonates with severe/acute medical or surgical conditions might have needs which are better fulfilled by US-guided CVCs.

NIR technology aids in locating viable superficial venous sites and in decreasing procedural time for short peripheral catheter insertion. However, its use in neonatal field is still a matter of debate. Theoretically NIR could give its best performance especially during the placement of ECCs [2].

One of the problems faced during the insertion of CVCs is to locate its tip position. Recently, it has been suggested to implement the real time technology to verify the tip location in order to reduce the risks of primary malposition such as the use of ultrasonography and intracavitary- electrocardiogram (IC-ECG) [3].

**Conclusions**

In order to achieve competence in these new advancements and to help neonatologists to fill these specific gaps, we organized a training course with both theoretical and hands-on sessions during the XXV Congress of the Italian Society of Neonatology called “*New methods of implantation and management of vascular access in neonatology”* accordingly to the recent recommendations of the WoCoVA.

**References**

1. M. Pittiruti. Ultrasound Guided Central Vascular Access in Neonates, Infants and Children. Current Drug Targets. 2012; 3:961-969.

2. M. Lamperti, M. Pittiruti. Difficult peripheral veins: turn on the lights. Br J Anaesth. 2013; 110:888–891.

3. A. Capasso, R. Mastroianni et al. The intracavitary electrocardiography method for positioning the tip of epicutaneous cava catheter in neonates: Pilot study. J Vasc Access. 2018;19:542-547.

## A23 Communication strategies in neonatology: how to improve the quality of the interventions, how to communicate bad news, how to talk about uncertainty

### Silvana Quadrino^1^, Alessandra Coscia^2^, Lorenzo Colombo^3^

#### ^1^Istituto CHANGE, Scuola di Comunicazione e counselling sistemico-narrativo, Turin, Italy; ^2^Università degli Studi di Torino, SCDU Neonatologia Azienda Ospedaliera OIRM-S.Anna, Turin, Italy; ^3^Fondazione IRCCS Cà Granda Ospedale Maggiore Policlinico, U.O. di Neonatologia e Terapia Intensiva Neonatale, Milan, Italy

##### **Correspondence:** Silvana Quadrino (silvana.quadrino@gmail.com)

**Background**

One of the difficulties that neonatologists have to face is the parents' expectation of birth as a "happy event". When it is necessary to give parents information that modifies this expectation, even if only slightly, the informational intervention requires attention and competence. When the change in expectations includes a risk for the child's future, for his health, for his survival, specific skills are needed to help parents to face the new situation and lay the foundations for a relationship based on trust and on the alliance between professionals and family.

**Method**

The course aims to bring participants closer to the method that the autors used in different departments of neonatal intensive care in Italy^i^. The method is based on a systemic approach and on the use of narrative medicine.^ii iii^It allows the doctor to improve the quality of the exchange of communications with the parents, to facilitate the explanation of their premises, their values, their requests, even starting from the cultural specificities. It also allows to identify the obstacles that can render the professional interventions ineffective and and to choose communication strategies to overcome them and to make good use of the available time.

The topics that will be addressed are:

1. Communication and information: to improve the effectiveness of information interventions and to avoid misunderstanding it is necessary to be aware of everything that can hinder the understanding of information, from emotional to cultural and linguistic obstacles, and to have techniques to overcome them.
  One of these techniques is the ability to turn informational communication from "one-way" (from professional to family) into "two-way" communication, based on the ability to ask questions that facilitate understanding and acceptance of informational messages.
2. The communication of bad news: everything that irreversibly alters people's expectations of their future must be considered bad news. It is not possible to avoid the emotional reactions that such news causes - pain, fear for the future, anger, etc. It is however possible to make them more tolerable, and to facilitate the family in facing the situation with ways compatible with its own specific characteristics.
3. The communication of uncertainty: when "something is wrong" the parents ask the professionals certainties that often cannot be given: what will happen in the next few hours, in the next days, how the child will be in the future, etc. It is necessary to learn to say "I don't know" without being brutal, without erasing hope and without compromising the parents' trust in professionals.

**Conclusions**

Physicians and nurses can improve the quality of their communications with patients and families through interactive training, which allows them to acquire the

basic techniques for information, for communicating bad news and for communicating uncertainty. In training interventions participants' experience is used, and practical cases are analyzed, deepened and transformed into communication exercises to practice with the proposed techniques.

^i^ C. Fabris, A. Coscia, E. Bertino, M. Prete, L. Occhi, F. Giuliani, S. Quadrino, Counselling in Neonatal Intensive Care Unit (NICU) in Early Human Development 85 (2009) S47–S48

^ii^ G.Bert Medicina narrativa, storie e parole nella relazione di cura, Il Pensiero Scientifico 2007

^iii^ S. Quadrino Il dialogo e la cura: le parole fra medici e pazienti , Il Pensiero Scientifico 2019

## A24 Impact of RSV infection in infants: biological basis of short and long term sequelae

### Giovanni A. Rossi, Oliviero Sacco, Donata Girosi, Michela Silvestri

#### Department of Pediatrics, Pediatric Pulmonary Disease Unit, IRCCS Istituto Giannina Gaslini, Genoa, Italy

##### **Correspondence:** Giovanni A. Rossi (giovannirossi@gaslini.org)

**Abstract**

Respiratory syncytial virus (RSV) is the most common agent of severe airway disease in infants and young children [1]. In early childhood, and even more in preterms, the physiological inefficiency of the immune system may lead to an inefficient Th1 adaptive response, excessive inflammation and severe damage to the fragile respiratory structures [2]. Prematurity is the leading risk factor associated with the severity of the lower respiratory tract involvement. In preterm infants, the lower efficiency of dendritic cells, the reduced number of circulating memory-helper-inducer T-cells and the inadequate activation of the cytotoxic CD8+ cells may all contribute to the increased susceptibility to severe RSV disease [2]. Moreover, the embryonic saccular phase (28-36 weeks’ gestational age) is a critical transitional period in which lung volumes rapidly increase, with full maturation of alveolar-capillary units that become efficient gas exchangers [3]. A clear relationship also exists between the severity of the first RSV infection and subsequent recurrent wheezing and asthma episodes [4]. This link is related to long-term changes in neuroimmune control of airway smooth muscle tone, rather than to an increased susceptibility to allergic sensitization [2]. RSV infection induces a neurogenic inflammation with upregulation of nerve growth factor (NGF), enhanced release of acetylcholine, neurokinin A and substance P (SubP), all involved in the regulation of bronchomotor tone [2,5]. NGF also promotes a long-term remodeling of the non-noradrenergic, non-cholinergic innervation in the respiratory tract, favoring the overgrowth of neurites with higher SubP content [2,5]. One mechanism underlying the respiratory sequelae of the early-life RSV can be the persistence of a latent viral infection that can maintain a constant stimulation of the immune system [6]. The short and long term sequelae of RSV infection may also be related to a genetic predisposition. However, positive associations between single nucleotide polymorphisms related to RSV severity and to the increased asthma risk have been only rarely confirmed when tested in different countries/populations [7]. Asthma risk may be increased by RSV-induced epigenetic modifications, as shown in animal models [8]. Finally, in young children, severe RSV infections can be complicated by invasive pneumococcal disease [9]. In *in vitro* studies, RSV facilitates bacterial super-infections, not only downregulating the innate and adaptive immune responses [2], but also enhancing the expression of adhesion molecules for *Streptococcus pneumoniae* on the bronchial epithelial surface*,* inducing tight junction disassembly on ciliated epithelial cells and increasing the nutrient availability in the airways [2,10,11].

**References**

1. Nair H, Nokes DJ, Gessner BD, Dherani M, Madhi SA, et al. Global burden of acute lower respiratory infections due to respiratory syncytial virus in young children: a systematic review and meta-analysis. Lancet 2010: 375: 1545–55.

2. Rossi GA, Colin AA. Respiratory syncytial virus-Host interaction in the pathogenesis of bronchiolitis and its impact on respiratory morbidity in later life. Pediatr Allergy Immunol. 2017; 28: 320-331.

3. Colin AA, McEvoy C, Castile RG. Respiratory morbidity and lung function in preterm infants of 32 to 36 weeks' gestational age. Pediatrics. 2010; 126: 115-28.

4. Régnier SA, Huels J. Association between respiratory syncytial virus hospitalizations in infants and respiratory sequelae: systematic review and meta-analysis. Pediatr Infect Dis J. 2013; 32: 820-6.

5. Li QG, Wu XR, Li XZ, Yu J, Xia Y, et al. Neural-endocrine mechanisms of respiratory syncytial virus-associated asthma in a rat model. Genet Mol Res. 2012; 11: 2780-9.

6. Rezaee F, Gibson LF, Piktel D, Othumpangat S, Piedimonte G. Respiratory syncytial virus infection in human bone marrow stromal cells. Mol Biol. 2011; 45: 277-86.

7. Larkin EK, Hartert TV. Genes associated with RSV lower respiratory tract infection and asthma: the application of genetic epidemiological methods to understand causality. Future Virol. 2015; 10: 883-897.

8. Ptaschinski C, Mukherjee S, Moore ML, Albert M, Helin K, et al. RSV-Induced H3K4 Demethylase KDM5B Leads to Regulation of Dendritic Cell-Derived Innate Cytokines and Exacerbates Pathogenesis In Vivo. PLoS Pathog. 2015; 11: e1004978.

9. Talbot TR, Poehling KA, Hartert TV, Arbogast PG, Halasa NB, et al. Seasonality of invasive pneumococcal disease: temporal relation to documented influenza and respiratory syncytial viral circulation. Am J Med 2005; 118: 285-291.

10. Avadhanula V, Rodriguez CA, Devincenzo JP, Wang Y, Webby RJ, Ulett GC, Adderson EE. Respiratory viruses augment the adhesion of bacterial pathogens to respiratory epithelium in a viral species- and cell type-dependent manner. J Virol. 2006; 80: 1629-36.

11. Hendricks MR, Lashua LP, Fischer DK, Flitter BA, Eichinger KM, Durbin JE, Sarkar SN, Coyne CB, Empey KM, Bomberger JM. Respiratory syncytial virus infection enhances Pseudomonas aeruginosa biofilm growth through dysregulation of nutritional immunity. Proc Natl Acad Sci U S A. 2016; 113: 1642-7.

## A25 Impact on hospital readmission rates of a new protocol for the analysis of cardiorespiratory stability in preterm infants before NICU discharge: The CORE Study

### Federica Runfola^1^, Francesca Carpano Maglioli^1^, Chiara Maddaloni^1^, Giulia Pruccoli^1^, Enrico Cocchi^1^, Elena Maggiora^2^, Federica Logrippo^2^, Maria Chiara Ariotti^2^, Francesca De Matteis^2^, Enrico Bertino^2^, Francesco Cresi^2^

#### ^1^Department of Pediatric and Public Health Sciences, University of Turin, Turin, Italy; ^2^Neonatal Intensive Care Unit, Department of Public Health and Pediatrics, University of Turin, Turin, Italy

##### **Correspondence:** Federica Runfola (federica.runfola@yahoo.it)

**Background**

Prematurity is a risk factor for hospital readmission in newborns, especially in the first years of life. This could be due in part to the lack of universally accepted criteria for neonatal intensive care unit (NICU) discharge. A standardized and objective approach to cardiac and respiratory evaluation may grant a safer discharge for patients at high risk.The purpose of this study is to assess the efficacy of the “Cardio Observation and Respiratory Evaluation” (CORE) protocol in reducing hospital readmissions after NICU discharge.

**Materials and methods**

**Fig. 1 (abstract A25).**
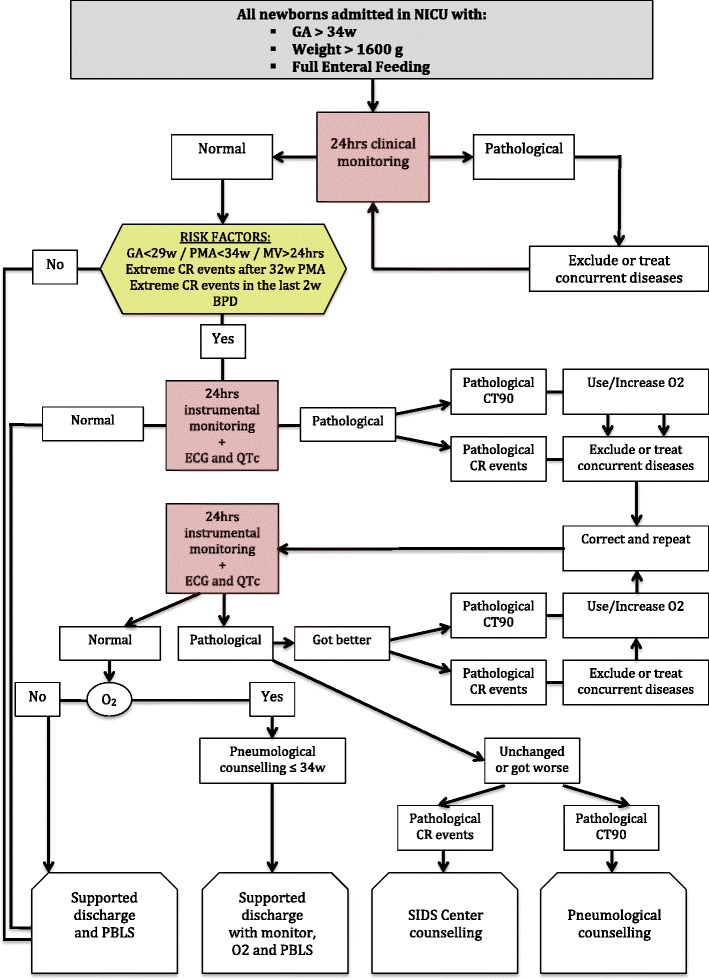
The CORE study flowchart. Abbreviations: NICU=Neonatal intensive care unit; W=weeks; GA=Gestational age; PMA=post-menstrual age; MV=mechanical ventilation; CR=cardio-respiratory; BPD=Bronchopulmonary dysplasia; CT90=Cumulative time percentage with SpO2 <90%; LOS=Length of stay; PBLS=Pediatric Basic Life Support; SIDS=Sudden infant death syndrome

A retrospective analysis was conducted on preterm infants (gestational age, GA: 25^+0^ - 33^+6^ weeks) born between November 2015 and January 2018 and managed by two independent medical-nursing teams at NICU of Sant’Anna Hospital in Turin. Patients in the case group (110) were evaluated with CORE before discharge and matched with a 1:2 ratio to controls, which were managed according to good clinical practice.The CORE protocol (Figure 1) is a stepwise approach.It consists of a screening phase before discharge, made up of a 24-hour clinical observation. In case of high risk patients the clinical monitoring is followed by a 24-hour instrumental assessment.High risk patients are defined as follows: GA<28weeks and/or post-menstrual age, PMA≤34 weeks and/or a history of mechanical ventilation >24-hour and/or need for supplemental oxygen and/or evidence of extreme cardiorespiratory events in the last two weeks. Patients who failed one of the two monitoring underwent clinical reassessment and were evaluated again one week later.The primary outcome of this study was hospital readmission rates during 12 months after discharge. The secondary outcome was the length of hospital stay (LOS) in NICU.

**Results**

We compared 110 cases (median GA 30.6 weeks, IQR 28.4–32.3) with 213 controls (median GA 30.9 weeks, IQR 28.7-32.3). The main features of the two groups were similar. Forty-seven patients (42,7%) from the cases matched high risk criteria and subsequently underwent instrumental monitoring. In this group we found a significant lower hospital readmission rate (log-rank p<0.05) compared to the controls. The difference was especially pronounced within 3 months of discharge (cases: 9.09% vs. controls: 21.6%; p: 0.004). Cases and controls had a NICU LOS of 39 (26-58) and 43 (26-68) days respectively (p: 0.16).

**Conclusions**

The use of CORE to evaluate cardiorespiratory stability in preterms is related to a lower hospital readmission rate, especially within 3 months of discharge, without increasing the LOS in NICU.

## A26 Update and drawning-up of a Protocol about the management and dressing of the central venous catheter (PICC) and the infusion lines after literature review

### Rita Sacco^1^, Claudia Recchia^2^, Andrea Parigiani^3^

#### ^1^Pediatric nurse - NICU of Policlinico Casilino, Rome, Italy; ^2^Nurse - NICU of Policlinico Casilino, Rome, Italy; ^3^Nurse - NICU of Policlinico Casilino, Rome, Italy

##### **Correspondence:** Rita Sacco (Saccorita5@gmail.com)

**Introduction**

The technique to insert a silicone catheter starting from a peripheral vein to get into the vena cava near the heart, was described for the first time in 1973.

P.I.C.C. (Peripherally Inserted Central Catheter) is the most common abbreviation of this device, better known in Italian with the abbreviation CVC (Central Venous Catheter).

Proper nursing management of the PICC, extends its durability and prevents all the possible and serious complications such as catheter-related systemic infections, peripheral phlebitis, venous thrombosis, cardiac thrombosis, local skin injuries.

Nowdays, however, there are lacking scientifically-validated protocols about the menagement of CVC and according to the most recent guidelines, every Care Units (CU) should draw up an internal protocol for the "PICC care".

After a review of the literature and than a debate between nurses of our NICU, we found some critical issues about the management of the CVC, pointing-out not univocal behavior on the following points:

1. How to do the CVC dressing;
2. When replacing the CVC dressing;
3. Management of the infusion line;
4. How to change the infusion line;

**Study Objective**

The aim of the study is to create and share, after the review of the most recent literature, a unique and updated protocol that would be able to standardise the management of the PICC within the NICU.

**Materials and methods**

The literature review was carried out through a research between different biomedical databases (using free keywords) and on the main search engines, consulting websites regarding preterm infants and nursing assistance in NICU.

**Results**

After the researches and the debate between the nurses of the CU, we have drawn up two protocols: the first one concerning the management of the CVC an the second one concerning the infusion line (Protocol A and Protocol B).

*Protocol A: Nursing management of the PICC dressing*

- Dressing after the catheter’s insertion.
- Materials for dressing
- Step-by-Step procedure
- PICC durability
- Reasons to replace the dressing

*Protocol B: nursing management of the infusion line*

- Procedures for maintenance of the infusion line
- Aseptic method of replacing infusion line

**Conclusions**

The periodic review of internal protocols performed by NICU’ staff is essential to point-out any mistakes in daily practice and represents an important moment for sharing and correcting doubts. In particular, a correct management of PICC is an essential factor for the intensive care nurse because it affects the prevention of possible serious risks associated with the permanence of CVC.

**Acknowledgements**

The most sincere acknowledgment goes to all the members of this research. They have dedicated a lot of time and all their knowledge for the project to take shape.

Thanks to the Head Physician Dr. Piermichele Paolillo and to the Dr. Simonetta Picone to have believed in our project and to have encouraged us to make "real" our idea.

Finally, the best acknowledgment goes to all the NICU’s members of the Policlinico Casilino.

Thanks to all the nurses, all the doctors and the head nurse which have allowed to be carried out the project in our Care Unit.

**References**

1. Ista E, van der Hoven B, Kornelisse RF, et al. “Effectiveness of insertion and maintenance bundles to prevent central-lineassociated bloodstream infections in critically ill patients of all ages: a systematic review and meta-analysis”. Lancet Infect Dis 2016; 16: 724-34.

2. Kaufman DA, Blackman A, Conaway MR, et al. “Nonsterile glove use in addition to hand hygiene to prevent late-onset infection in preterm infants: randomized clinical trial”. JAMA Pediatr 2014; 168:909-16.

3. Cleminson J, Austin N, McGuire W. "Prophylactic systemic antifungal agents to prevent mortality and morbidity in very low birth weight infants". The Cochrane Library 2015.

4. Capretti M.G., Sandri F., Tridapalli E., Galletti S., Petracci E., Faldella G. “Impact of a standardized hand hygiene program on the incidence of nosocomial infection in very low birth weight infants”. Am J Infect Control, 2008; 36:430-435.

5. Marschall J., Mermel L.A., Classen D., Arias K.M., Podgorny K., Anderson D.J., Burstin H., Calfee D.P., Coffin S.E., Dubberke E.R., Fraser V., Gerding D.N., Griffin F.A., Gross P., Kaye K.S., Klompas M., Lo E., Nicolle L., Pegues D.A., Perl T.M., Saint S., Salgado C.D., Weinstein R.A., Wise R., Yokoe D.S. “Strategies to prevent central line-associated bloodstream infections in acute care hospitals”. Infect Control Hosp Epidemiol. 2008; 29: S22-S30.

6. Fox M, Molesky M, Van Aerde JE, et al. “Changing parenteral nutrition administration sets every 24 h versus every 48 h in newborn infants”. Can J Gastroenterol 1999; 13:147-151

## A27 Multicenter observational study on performance of blood sampling in neonatal population

### Gabriele Sorrentino^1^, Laura Panigada^2^, Alessia Fella^2^, Paola Marchisio^1,3^, Patrizio Sannino^4^, Serena Rampini^4^, Anna Lavizzari^1^, Laura Plevani^1^, Elena Nicoletta Bezze^1^, Fabio Mosca^1,5^

#### ^1^Foundation IRCCS Ca' Granda Ospedale Maggiore Policlinico, Milan, Italy; ^2^Studentessa del Corso di Laurea in Infermieristica Pediatrica, Università degli Studi di Milano, Fondazione IRCCS Cà Granda Ospedale Maggiore Policlinico, Milan, Italy; ^3^University of Milan - Department of Pathophysiology and Transplantation, Milan, Italy; ^4^Fondazione IRCCS Ca’ Granda Ospedale Maggiore Policlinico, Milan, Italy, Direzione Professioni Sanitarie; ^5^University of Milan - Department of Clinical Sciences and Community Health, Milan, Italy

##### **Correspondence:** Gabriele Sorrentino (gabriele.sorrentino@policlinico.mi.it)

**Background**

Blood sampling is one of the most common invasive procedures in neonatal units [1]. Blood specimens can be obtained for diagnostic and therapeutic purposes by direct venous, arterial or capillary blood sampling or by using a central venous catheter [2,3]. However, there is a paucity of studies aiming at identifying the most effective and safe method to collect blood samples in newborn infants.

The aim of the present study was to investigate the most frequently used methods for blood sampling in different Neonatal wards and Neonatal Intensive Care Units (NICU) in Lombardy, Italy.

**Materials and methods**

We conducted a multicenter, cross-sectional survey in 21 Neonatal Units in Lombardy from July 2018 to October 2018. Two questionnaires were developed [2-5]. The first questionnaire was administered to the head nurses of each unit to identify the organizational set-up; the second one was distributed to pediatric nurses/nurses to evaluate the following areas: general information for research participants, figures performing blood sampling, methods to collect blood specimens, material used for collecting a blood sample, skin disinfection practice, collection and transport of blood specimens and pain management. Data were processed applying descriptive analysis.

**Results**

A total of 33 head nurses and 395 pediatric nurses/nurses filled out the questionnaires. Among the participating centers, the number of admitted newborns was ≤ 2499 per year in 13 and > 2500 per year in 8, respectively. A written procedure describing the method for blood sampling within the department was present in 12 centers. Pediatric nurses/nurses were the principal professionals who performed venous, capillary and catheter blood sampling collection (71.9%, 96.2%, 74.3%, respectively). The most used method for venous blood sampling was “open system” composed by a butterfly (23 G) connected to a syringe. 75.8 % of healthcare professionals used chlorhexidine gluconate in alcohol-based to disinfect the skin. At least one non-pharmacological technique was used by healthcare professionals to collect a blood sample.

**Conclusions**

Although capillary blood sampling is a painful procedure, it remains the most common method to collect blood samples in newborn infants [1,4]. Those data are consistent with the literature [1,4]. Our survey showed that participating centers differ in terms of method to perform blood sampling, materials used and skin disinfection technique. Further studies to identify the most effective and safe method for both newborn infants and professionals are warranted.

**Acknowledgements**

We would like to thank the participating Neonatal Units: Fondazione IRCCS Ca’ Granda Ospedale Maggiore Policlinico (Milan), ASST Fatebenefratelli Sacco (Macedonio Melloni, Vittore Buzzi, Sacco), Fondazione Poliambulanza - Istituto Ospedaliero (Brescia), ASST Lecco - Ospedale Alessandro Manzoni, ASST Vimercate, ASST Grande Ospedale Metropolitano Niguarda (Milan), ASST Santi Paolo e Carlo (San Paolo), ASST Ovest milanese (Legnano, Magenta), ASST rhodense (Rho, Garbagnate), ASST nord milanese (Sesto San Giovanni), ASST Melegnano e della Martesana (Vizzolo Predabissi), ASST Lodi, ASST della Valle Olona (Gallarate, Saronno, Busto Arsizio), ASST Mantova, ASST Crema.

**References**

1- Carbajal R, Rousset A, Danan C et al. Epidemiology and treatment of painful procedures in neonates in intensive care units. JAMA. 2008; 300:60-70

2- Sironi C, Baccin G. Procedure per l’assistenza infermieristica. Milano: Masson; 2006

3- Lowe G, Stike R, Pollack M et al. Nursing blood specimen collection techniques and hemolysis rates in an emergency department: analysis of venipuncture versus intravenous catheter collection techniques. J Emerg Nurs. 2008; 34:26-32

4- Janes M, Pinelli J, Landry S et al. Comparison of capillary blood sampling using an automated incision device with and without warming the heel. J Perinatol. 2002; 22:154-8

5- O'Connor C, Philip RK, Powell J et al. Combined education and skin antisepsis intervention for persistently high blood-culture contamination rates in neonatal intensive care. J Hosp Infect. 2016; 93:105-7

## A28 Side effects associated with the implementation of current guidelines for parenteral nutrition in very preterm newborns

### Giovanni Boscarino, Francesca Faccioli, Elisa Onestà, Gianluca Terrin

#### Neonatal Intensive Care Unit, Department of Maternal and Child Health Policlinico Umberto I, University La Sapienza, Rome, Italy

##### **Correspondence:** Gianluca Terrin (gianluca.terrin@uniroma1.it)

**Background.** Current guidelines for parenteral nutrition (PN) in very low birth weight (VLBW) infants recommend a high protein and energy intake to limit extra-uterine growth retardation (EUGR) [1]. Enhanced parenteral nutrition (EnPN) improves the nitrogen balance of stable preterm newborns. However, recent evidences suggested that EnPN represents a burden for the metabolism of critically ill subjects, increasing the risk of morbidity and mortality [2]. We systematically revised and analyzed the available evidence to assess the safety of EnPN in preterm newborns. **Methods.** As data source we used Pubmed and ISI web of Knowledge databases, using the following subject headings and terms: parenteral nutrition, preterm newborn, nutritional intake, protein intake, energy intake. We selected and analyzed studies comparing EnPN vs. standard PN or vs. other nutritional approach in regard of the occurrence of side effects associated with PN. **Results.** We selected 23 studies, 16 RCT and 7 Cohort study (Table 1). Only 9 out of 16 RCT and 5 out of 7 Cohort study have evaluated side effects associated to PN. Two studies reported an association between hyperglycemia and EhPN, while 3 RCT demonstrated an increased risk of hyperglycemia in newborn receiving standard PN. An increased risk of hypertriglyceridemia in newborns receiving EnPN was reported by 1 RCT. One study demonstrated an increased risk of hypercalcemia and hypophatemia in newborn receiving EnPN. One study reported an increased risk of metabolic acidosis for EnPN nourished newborns. **Conclusion.** Consistent number of available studies reported an increased risk of side effects associated with the implementation of recent Guidelines on PN [1]. Thus, we call for caution in regard of EnPN use for preterm. Further studies are advocated to verify safety of aggressive nutritional strategies particularly in critically ill VLBW newborns.

**Acknowledgements**

None.

**References**

1. van Goudoever JB, Carnielli V, Darmaun D, Sainz de Pipaon M, Braegger C, Bronsky J, et al. ESPGHAN/ESPEN/ESPR/CSPEN guidelines on pediatric parenteral nutrition: Amino acids. Clin Nutr. 2018 Dec;37(6 Pt B):2315-2323.

2. Vanhorebeek I, Verbruggen S, Casaer MP, Gunst J, Wouters PJ, Hanot J, et al. Effect of early supplemental parenteral nutrition in the paediatric ICU: a preplanned observational study of post-randomisation treatments in the PEPaNIC trial. Lancet Respir Med. 2017 Jun;5(6):475-483.

**Table 1 (abstract A28).**
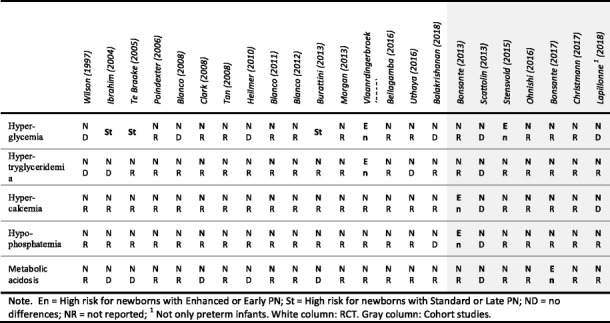
RCT and Cohort studies comparing enhanced vs. standard parenteral nutritional protocols in very low birth weight newborns

Note. En = High risk for newborns with Enhanced or Early PN; St = High risk for newborns with Standard or Late PN; ND = no differences; NR = not reported; ^1^ Not only preterm infants. White column: RCT. Gray column: Cohort studies

